# Proton Radiation Therapy: A Systematic Review of Treatment-Related Side Effects and Toxicities

**DOI:** 10.3390/ijms252010969

**Published:** 2024-10-11

**Authors:** Peter P. Salem, Perla Chami, Remy Daou, Joseph Hajj, Haibo Lin, Arpit M. Chhabra, Charles B. Simone, Nancy Y. Lee, Carla Hajj

**Affiliations:** 1 Faculty of Medicine, American University of Beirut, Beirut 1107, Lebanon; pps05@mail.aub.edu (P.P.S.); pnc00@mail.aub.edu (P.C.); 2 Family Medicine Department, Hotel Dieu de France Hospital, Beirut 1660, Lebanon; remy.daou@dbayehmedical.com; 3 Faculty of Medicine, University of Balamand, Beirut 1100, Lebanon; joseph.hajj@std.balamand.edu.lb; 4 New York Proton Center, New York, NY 10035, USA; hlin@nyproton.com (H.L.); achhabra@nyproton.com (A.M.C.); simonec1@mskcc.org (C.B.S.II); leen2@mskcc.org (N.Y.L.); 5 Memorial Sloan Kettering Cancer Center, New York, NY 10027, USA

**Keywords:** proton radiation therapy, neurotoxicity, pulmonary toxicity, cardiac toxicity, gastrointestinal toxicity, hematological toxicity, urological toxicity, osteological toxicity, oral toxicity, endocrine toxicity

## Abstract

Cancer is the second leading cause of death worldwide. Around half of all cancer patients undergo some type of radiation therapy throughout the course of their treatment. Photon radiation remains (RT) the most widely utilized modality of radiotherapy despite recent advancements in proton radiation therapy (PBT). PBT makes use of the particle’s biological property known as the Bragg peak to better spare healthy tissue from radiation damage, with data to support that this treatment modality is less toxic than photon RT. Hence, proton radiation dosimetry looks better compared to photon dosimetry; however, due to proton-specific uncertainties, unexpected acute, subacute, and long-term toxicities can be encountered. Reported neurotoxicity resulting from proton radiation treatments include radiation necrosis, moyamoya syndrome, neurosensory toxicities, brain edema, neuromuscular toxicities, and neurocognitive toxicities. Pulmonary toxicities include pneumonitis and fibrosis, pleural effusions, and bronchial toxicities. Pericarditis, pericardial effusions, and atrial fibrillations are among the cardiac toxicities related to proton therapy. Gastrointestinal and hematological toxicities are also found in the literature. Genitourinary toxicities include urinary and reproductive-related toxicities. Osteological, oral, endocrine, and skin toxicities have also been reported. The side effects will be comparable to the ones following photon RT, nonetheless at an expected lower incidence. The toxicities collected mainly from case reports and clinical trials are described based on the organs affected and functions altered.

## 1. Introduction

Cancer is the second leading cause of death worldwide after cardiovascular disease. According to the International Agency for Research on Cancer, around 18.1 million cancer cases were recorded in the year 2020. Nearly 10 million deaths in 2020 were cancer-related, and this figure is predicted to increase to 16 million in the upcoming 20 years [1,2]. Radiation therapy remains one of the most effective neoplastic treatment options, with approximately half of cancer patients estimated to receive some type of radiation therapy as part of their treatment [3]. Radiation damages the cell’s genetic material, leading to its death or inability to undergo cell division. Hence, by depositing energy into the cell, radiation ultimately prevents its proliferation and leads to cell death [4].

The conventional radiotherapy modality is photon radiation; however, recent advancements in proton therapy have shown promising and better results [5]. Unlike photons, when protons penetrate matter, they slow down with depth until they reach an abrupt stop where most of their energy is deposited; this is known as the Bragg peak and serves as a key characteristic in proton radiation [6,7]. This allows the sparing of healthy tissue from the effects of radiation along the proton beam’s entry and exit line [7]. In clinical practice, protons have been assumed to have a 10% increase in biological effectiveness when compared to photons [6]. Hence, the Relative Biological Effectiveness (RBE) of protons is set to 1.1 when computing the effective doses from physical doses in Gray [7]. Throughout the years, proton radiation has improved its treatment planning and delivery methods to increase effectiveness and reduce toxicities. Passively scattered proton therapy (PSPT) utilizes a simple design of a rotating modulation wheel as well as one or two scatters to manipulate the proton beam laterally and longitudinally according to the target volume [7]. Scanning beam proton therapy (SBPT) utilizes an energy degrader to fine-tune beam energy and magnets to sweep the proton beam laterally, accurately filling the target volume [6,8]. Intensity-modulated proton therapy (IMPT), a more advanced form of proton therapy, utilizes pencil beam scanning (PBS) technology to optimally modulate dose distributions to achieve high dose conformity to target volume and maximize the protection of normal organs [9]. Proton radiation has shown promising advances in the field of radiotherapy. However, the high sensitivity of proton delivery to range uncertainties, beam path variations, and organ motion may lead to unexpected hot and cold dose points in treatment. In addition, the combination of the enhanced RBE at the distal end of the beam and range uncertainties may result in dose delivery uncertainties. Some unfavorable outcomes and unexpected toxicities are being reported [6] due to these uncertainties.

In this paper, we present a comprehensive review of the underreported and/or unexpected side effects, as well as expected acute, subacute, and long-term toxicities that have been described in patients undergoing treatment with proton beam therapy (PBT). This review evaluates different toxicities collected from the existing literature on the basis of organs and functions affected. The term underreported is used when comparing toxicities from proton radiotherapy to photon radiotherapy, indicating that far less information regarding this toxicity is found in association with proton radiotherapy in comparison to photon radiotherapy.

## 2. Materials and Methods

**Figure 1:**
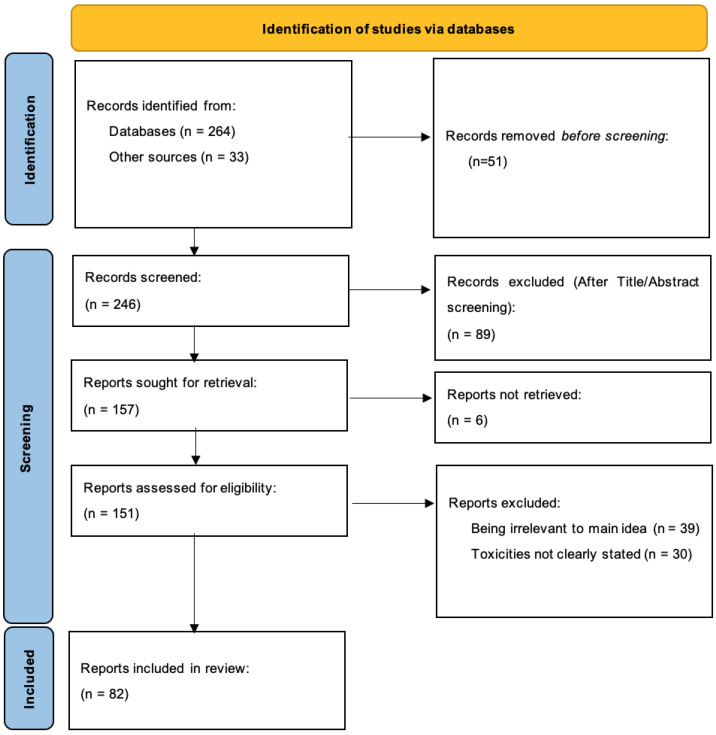
PRISMA flow diagram depicting the search strategy in the systematic review literature search.

This systematic review was conducted in accordance with the Preferred Reporting Items for Systematic Reviews and Meta-Analysis (PRISMA) guidelines. Two authors (P.P.S. and P.C.) independently conducted the review using PubMed. There was no disagreement between the authors regarding the eligibility of selected studies. The search covered a period from 1993 to 2024. An advanced search strategy was followed using key words in the following way: ((proton) AND (radiation) AND (toxicity)), and filters for the inclusion of case reports, clinical trials, and randomized control trials were used when possible. The aforementioned is shown in Figure 1.

The eligibility criteria were case reports and prospective and retrospective case series that effectively reported patient outcomes regarding radiation toxicity following proton radiation treatment. The exclusion criteria were review articles, meta-analyses, and studies that do not effectively report radiation toxicity. Some papers were identified by scanning the references of relevant papers. This review includes 3 randomized control trials.

## 3. Results

### 3.1. Proton Radiation Therapy (PBT)

**Figure 2:**
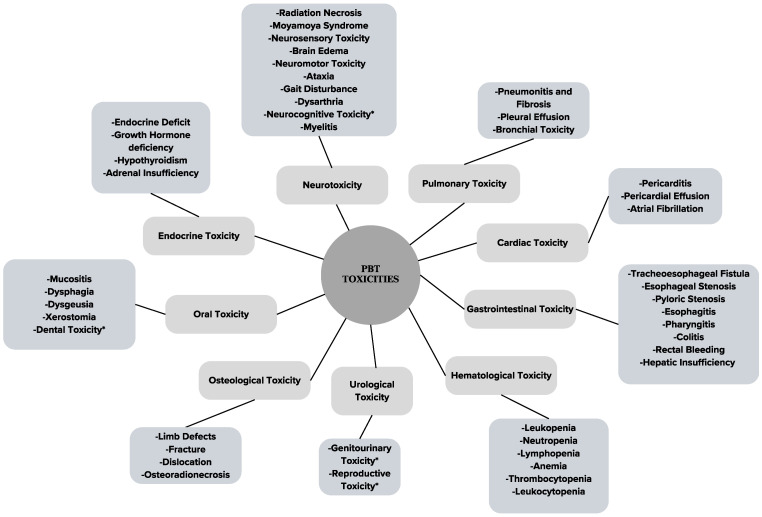
Summary of proton radiation therapy (PBT) toxicities. * Further expanded in manuscript.

PBT makes use of protons’ physical properties to precisely deliver conformal radiation doses in target regions sparing healthy tissue, using energy and time modulation of protons’ Bragg peak [7]. The main setbacks of this modality generally include high costs and technological accessibility when it comes to treatment planning [10]. PBT may result in treatment-related toxicities that will be discussed as summarized in Figure 2.

### 3.2. Neurotoxicities

#### 3.2.1. Radiation Necrosis

Brain tissue necrosis is a serious complication that arises from radiotherapy of intracranial and skull base tumors. Radiation triggers cell death and apoptosis through interactions with the cytoplasmic membrane, production of reactive oxygen species, and DNA damage [11]. Radiation necrosis (RN) presents as endothelial damage, gliosis, and perilesional edema found mainly in white matter [12]. Reports suggest the incidence of radiation necrosis in cases of primary and metastatic brain tumors with an incidence rate of 3–24% following focal radiotherapy [13,14]. In general, subsequent chemotherapy, radiation dose, and fraction size are risk factors for radiation necrosis [15]. PBT has been linked to incidences of radiation-induced brain necrosis. A retrospective study conducted on 20 patients treated with fractionated PBT for recurrent gliomas between the years 2005 and 2012 recorded two cases (10%) of long-term radiation-induced necrosis. Of these two cases, a 27.3-year-old patient having recurrent GBM was diagnosed 8.5 months after treatment, and a 58.4-year-old patient having recurrent anaplastic (grade 3) oligodendroglioma was diagnosed 63 months after treatment [16]. Kalapurakal et al. report the incidence of three cases (21%) of radiation necrosis in a study discussing the preliminary results of intraoperative photon radiotherapy in children with recurrent brain tumors [17]. A prospective study conducted between 2008 and 2020 by the UK National Health Service, reported clinical outcomes for patients having central nervous system (CNS), base of skull, spinal, and paraspinal tumors treated with PBT. Of the 702 evaluable patients in the cohort, 2 cases of grade 5 CNS necrosis were reported. The authors attribute the prevalence of these unexpected severe toxicities to RBE uncertainties; they also mention that posterior fossa tumors have shown similar rates of brainstem toxicities when compared with photon series [18]. Moreover, RN was diagnosed 12 months after spot scanning proton therapy after three previous non-proton radiation treatments in a clinical case with a five-time recurrent orbital hemangiopericytoma. This patient’s RN was resolved through steroid and bevacizumab treatment. After undergoing another proton treatment for a fifth relapse, the patient unexpectedly died a year later. Pathology reports showed a brain abscess in the left hemisphere and ruled the adverse event not related to irradiation; however, the authors assume that this occurred due to an indirect treatment side effect since skull base bones exceeded their cumulative dose tolerance, which could have potentially led to the abscess and infection [19]. Also, Bronk et al. report the case of a 43-year-old man having grade II meningioma with residual disease after craniotomy and resection 13 years prior. After treatment with passive-scatter PBT, MRI demonstrated brain lesions suggestive of RN 14 months after PSPT [20]. Furthermore, a comparative study aiming to assess outcomes for patients after receiving proton or photon radiotherapy before stem cell transplant reported the incidence of CNS necrosis 10 months after proton craniospinal irradiation. Gunther et al. provide a statistical comparison showing no significant difference in the occurrence of neurotoxicity for proton versus photon CSI (*p* = 0.38) [21].

Radiation-induced brainstem toxicity can be defined as a new or developed injury to cranial nerves V–VII and IX–XII, motor weakness, and dysmetria [22]. The incidence of proton-induced brainstem toxicity has been quantified to be within a broad range, 0.7–16%, in pediatric patients, which prompts more rigorous efforts in reporting outcomes following proton irradiation which will be discussed further in the conclusion [23,24,25]. Proton therapy offers increased dose conformity and targeted dose distribution compared to photon therapy; patients with brain or skull base tumors are not without risk of developing brainstem necrosis. In a retrospective study looking to examine the incidence of brainstem toxicity in pediatric patients with brain or skull base malignancies following proton therapy, 11 of 313 patients developed brainstem toxicity within 2–12 months, and the cumulative 2-year incidence of grade 3+ toxicity was 2.1%. Moreover, a 7-year-old girl who received a prescription dose of 59.4 CGE that was in accordance with protocol guidelines suffered from an acute brainstem stroke 4 months after treatment. The authors mention that brainstem toxicity in pediatric patients having received photon radiation treatments is somewhere between 2% and 18%, so the following proton trial’s toxicity percentage lies within this range [24]. A more recent study aimed to evaluate the incidence of brainstem toxicity following proton therapy in 163 adult patients with skull base tumors and reported a 5-year cumulative incidence (grade 2 or higher) of brainstem toxicity of 1.3% [26]. Another study, which looked at the incidence of brainstem necrosis following PBS in pediatric patients with central nervous system tumors, found that of 166 patients, no patient experienced brainstem necrosis for first-line radiotherapy, with 1 patient who had undergone previous photon-radiation developing brainstem necrosis [27]. Gordon et al. evaluate the toxicity outcomes in a single-center study of patients treated with PBT for chordomas and chondrosarcomas. A grade 5 brainstem toxicity was experienced by 1 patient out of 31; however, this was predictable due to the extent of brainstem infiltration and the poor condition of this patient prior to treatment. It is worth mentioning that the tolerance dose was exceeded slightly as well [28]. In a multicenter prospective registry report from the Proton Collaborative Group, among 100 patients treated for chordoma, including 61 patients with primary base-of-skull tumors, 1 case of grade 2 CNS necrosis was reported, with no late grade 3+ events [29]. The incidence of proton-radiation-induced brainstem toxicity is comparable to that of photon radiation; however, as previously mentioned, more efforts should be made in quantifying its incidence as large-scale studies are lacking in the literature.

#### 3.2.2. Moyamoya Syndrome

Moyamoya syndrome (MMS) is considered a rare late complication and has been linked to treatment with radiotherapy, especially in pediatric patients below 5 years of age with tumors in the parasellar region [30]. It is a bilateral cerebrovascular disorder characterized by the obstruction or stenosis of the terminal section of the internal carotid arteries, resulting in reduced blood flow, apparent through an abnormal vascular network at the base of the brain [31]. Its incidence ranges between 3.5 and 20% [32]. Although PBT allows for increased dose conformity and minimal energy loss compared to photon radiotherapy, it was also shown to be associated with MMS. In a retrospective study looking to evaluate treatment outcomes in children with central nervous system germ cell tumors after PBT, a 5-year-old patient with a tumor in the suprasellar region of the brain was diagnosed with MMS 3 years post-treatment [33]. Moreover, in a recent case report, a 5-year-old child diagnosed with clival chordoma underwent treatment with PBT due to disease relapse 33 months after surgical resection and was found to have developed MMS a year later [34]. In another case study, a child with a medical history of ependymoma, close to the suprasellar region of the brain, had previously undergone near-total resection of the tumor at 7 months. The patient was then treated with chemotherapy followed by PBT 4 months after and was diagnosed with MMS at 2 years old [35]. Similarly, a 2.5-year-old boy with a low-grade astrocytoma, treated initially with chemotherapy, which was then followed by PBT due to radiographic progression, developed MMS 2 years post-PBT. In the same case series, a 5-year-old girl diagnosed with a craniopharyngioma presented with MMS 2 years after PBT treatment [36]. The incidence of radiation-induced toxicities in pediatric patients with brain tumors is believed to be due to irradiation at a time of rapid brain growth [37]. A systematic review study looking at the incidence of cerebral vasculopathy (including MMS) in pediatric tumors following treatment with photon or proton therapy reported a higher incidence of vasculopathy (6.3–20%) with photon radiation in comparison with PBT (5–9.9%) [38]. Another study has shown that patients receiving PBT for brain tumors are at an increased risk of developing MMS, with an incidence comparable to that of photon radiation and even higher. Therefore, MMS should be considered as a potential complication following PBT [39].

#### 3.2.3. Neurosensory Toxicities

Phthisis bulbi is one of the rarely reported eye-related radiation toxicities. It is characteristic of end-stage ocular disease, which leads to an atrophied and shrunken eye with disorganized intraocular structures, rendering it nonfunctional [40]. The following cases were the only ones in our search that mentioned the development of phthisis bulbi after PBT. In a recent case study, a patient diagnosed with bilateral retinoblastoma was treated with PBT, which resulted in phthisis bulbi in the right eye requiring enucleation nearly two years post-radiation [41]. In another case series, three patients diagnosed with choroidal melanoma underwent treatment with PBT, one of which had to undergo enucleation of the eye two years later due to phthisis bulbi [42].

Moreover, a study compared histopathologic and radiologic findings between PBT-treated uveal melanoma patients who underwent secondary enucleation and those who underwent primary enucleation without prior irradiation. Proton-radiation-induced necrosis had large and confluent necrotic foci and showed hyperintense T1-weighted images and hypointense T2-weighted images. This differs from the multiple small distinct foci seen on histopathology, and the hyperintense T2 with hypointense T1-weighted images appreciated on magnetic resonance imaging (MRI) in non-irradiated uveal melanomas. The authors state the importance of recognizing these changes to improve radiotherapeutic outcomes following PBT, particularly with follow-up MRI examinations [43,44].

More commonly reported PBT-induced eye-related toxicities include cataracts, retinopathy, optic neuropathies, neovascular glaucoma, and vision loss [45,46,47,48].

As for olfactory toxicities, our search did not divulge many incidences of phantosmia, which has been underreported following treatment with proton therapy. Phantosmia is a type of qualitative olfactory disorder that manifests through the perception of often unpleasant odors in the absence of a stimulus [49]. A retrospective study looking at the incidence of phantosmia in pediatric, adolescent, and young adult patients undergoing treatment with IMPT for primary intracranial, metastatic intracranial, skull base, nasopharyngeal, or sinonasal tumors reported 12 cases among 99 patients in which patients experienced mild or moderate phantosmia during and/or after radiation [50]. Moreover, severe phantosmia was reported in a case study of a 16-year-old patient diagnosed with myxopapillary ependymoma while undergoing proton therapy requiring general anesthesia [51].

Ototoxicity, which can manifest as tinnitus, hearing impairment, and/or vertigo has also been associated with proton therapy [52]. In a recent cohort study assessing toxicities of proton therapy in patients with vestibular schwannoma, 42% of patients developed progressive hearing loss within 1 year post-radiation, and 73% of patients with longer follow-up periods [53]. Similarly, in a study aiming to assess outcomes of pediatric patients diagnosed with ependymoma following treatment with proton therapy, 6.1% of patients experienced new hearing loss, which required the fitting of hearing aids [54]. In another study looking to evaluate the feasibility of PBT through the analysis of overall survival and local control rates in patients with chordoma and chondrosarcoma of the skull base, 6 patients of 18 developed grade 2 otitis media, of which 3 experienced persistent otitis for over three months [55]. Moreover, chronic recurrent otitis media as a result of PSPT-induced eustachian tube dysfunction was reported in 2 patients out of 29 undergoing treatment for nonmetastatic chordoma [56]. However, grade 3 and 4 ototoxicity rates were found to be similar in one study comparing ototoxicity rates in patients with medulloblastoma treated either with photons (*n =* 46) or PSPT (*n* = 38), even though patients in the proton group received lower cochlear doses. On the common terminology criteria for adverse events (CTCAE) scale, grade 3 and 4 ototoxicity was reported in 28.3% of patients undergoing photon radiation and 29.9% of patients undergoing proton radiation (*p* = 1.0) [57].

#### 3.2.4. Brain Edema

Brain edema is the unusual accumulation of fluids within the brain parenchyma [58]. It is divided into two types: vasogenic brain edema occurs when the origin of this fluid is the surrounding blood vessels and is due to disruptions in the blood–brain barrier, and cytotoxic brain edema occurs when fluid and sodium ions build up within the cells due to injury. This leads to swelling and a life-threatening increase in intracranial pressure [58,59,60]. Brain edema has been reported as a side effect of conventional radiation therapy, and PBT has also been linked to incidences of brain edema [60]. Greenfield et al. discuss the results of a prospective study where 20 patients were assessed after receiving proton radiation treatments for germinomas or non-germinomatous germ cell tumors (NGGCTs). Of the 20 patients, 4 were reported to have faced acute-onset brain edema requiring medical intervention [33]. Furthermore, another prospective study reported a case of late grade 4 cerebral edema in a patient receiving fractionated combined proton–photon radiotherapy for benign meningioma [61]. Brain edema following radiotherapy is a common occurrence, usually being asymptomatic. A study reported that 40% of the 200 enrolled patients developed asymptomatic brain edema at the area of maximum dose [60].

#### 3.2.5. Neuromotor/Neuromuscular

Radiation therapy has long been linked to negative effects on fine motor movement coordination and executive function [62]. Reported outcomes of neuromuscular and neuromotor deficits exist in the literature. Out of 44 analyzed patients enrolled in a prospective trial for the treatment of benign meningioma, 1 patient was treated with combined proton–photon radiotherapy and faced severe grade 4 neuromotor deficits. The authors hypothesize that the grade 2+ toxicities in their study might have been due to exceeding the 54 GyRBE dose constraint for intracranial critical structures [61]. More specific toxicities arising from proton-related treatments are discussed below.

#### 3.2.6. Ataxia

Ataxia is the reduced coordination of voluntary muscle movements. It usually arises due to dysfunction in the cerebellum or diminished proprioceptive or vestibular input to the cerebellum [63]. Upadhyay et al. report ataxia after PBT in pediatric tumors treated at MD Anderson Proton Therapy Center between 2007 and 2019. Of the 468 evaluable patients, 5 patients with ependymomas were diagnosed with ataxia. The death of a patient with ataxia was due to respiratory failure related to the brainstem injury that caused ataxia. Another patient initially diagnosed with medulloblastoma developed ataxia post-treatment. Four other patients with atypical teratoid rhabdoid tumors were diagnosed with ataxia [64]. A prospective study conducted to analyze outcomes of patients with recurrent glioblastoma after PBT reported the incidence of grade 3 ataxia in 1 patient out of 45. Compared to photon trials, this trial showed comparable results [65]. Another prospective trial with 67 evaluable patients by the end of the study compared outcomes of newly diagnosed glioblastoma patients after receiving either proton radiotherapy or intensity-modulated radiotherapy. One patient enrolled in this trial had grade 1 ataxia after proton radiotherapy treatment, and three patients in the IMRT arm had grade 1–2 ataxia [66]. Moreover, Sanford et al. present toxicity outcomes from a prospective trial where 44 patients were treated with combined fractionated proton–photon radiotherapy for benign meningiomas, in which two patients developed grade 1 ataxia and two other patients developed grade 2 ataxia post-treatment [61]. Ataxia incidence is related to systemic brainstem injury (SBI). Several studies have observed a reduced risk of SBI with PBT when Dmax < 55.8 Gy RBE and V55 ≤ 6% [23].

#### 3.2.7. Gait Disturbance

Gait disturbances correspond to any deviations from normal walking in terms of posture, pace, stance, and other factors [67]. These disturbances arise from several etiologies, with one being neurological damage after radiation therapy. PBT has been rarely reported to have some effect on the gait of patients post-treatment. Yang et al. report a case out of cohort 20 patients of grade 2 acute gait disturbance in a patient treated with hypofractionated proton CSI for leptomeningeal metastases [68]. No relation between gait disturbance incidence/severity and radiation dose can be made based on the available scarce literature.

#### 3.2.8. Dysarthria

Dysarthria is a speech disorder in which the muscles of the mouth are affected, resulting in slurred speech and the inability to produce recognizable words. Dysarthria can be caused by neurological damage or brain injury [69]. Dysarthria has been reported in the literature after radiation to the brain [70]. There have also been some cases of dysarthria recorded after treatment with PBT. A prospective study aiming to analyze treatment toxicities of patients treated with proton therapy for low-grade gliomas reported late dysarthria in 1 out of 20 patients [71]. Furthermore, in the previously discussed prospective study by Sanford et al., where benign meningioma was treated with combined fractionated proton–photon radiotherapy, 1 patient out of 44 suffered from late-onset dysarthria [61].

#### 3.2.9. Neurocognitive

Despite the potential benefits of craniospinal irradiation, it has been known to cause deleterious cognitive deficits. The mechanism of cognitive disturbance following radiotherapy involves oligodendrocyte depletion, vascular injury, and neurogenesis inhibition [72]. Deficits in memory, attention, language, and executive functions are potential morbidities after radiation to the brain [73]. Moreover, conventional radiotherapy has been shown to have negative cognitive effects in a dose-dependent manner [74]. Although relatively underreported, PBT has also shown negative cognitive effects in the literature. One event of grade 1 cognitive deficit was reported in a prospective trial evaluating 20 patients treated with hypofractionated PBT for low-grade gliomas [71]. In a previously discussed prospective trial of 44 evaluable patients, 13 patients reported grade < 4 late-onset cognitive deficits, and 4 patients reported grade < 2 acute-onset cognitive deficits [61]. Furthermore, Gunther et al. reported a case of a patient clinically demonstrating late-onset neurocognitive impairment after CSI with protons and a stem cell transplant for a hematologic malignancy [21]. Another prospective trial assessing the adverse effects of PBT on patients diagnosed with pediatric rhabdomyosarcoma reported a case of grade 2 cognitive disturbance in 1 patient out of the 57 enrolled patients [75]. Saeed et al. reported a case of late-onset grade 3 cognitive disturbance in 1 patient out of 45 with recurrent glioblastoma enrolled in the Proton Collaborative Group Study 01-009 trial and treated with PBT [65]. Also, out of 702 patients, 9 cases of grade 3 cognitive disturbance were reported in patients with brain tumors enrolled in the UK National Health Service’s study aiming to assess the safety of PBT [18]. Other common neurocognitive/neurobehavioral toxicities after PT include anorexia, vertigo, and depression [66,68]. In a comparison of neurocognitive toxicity between proton and photon therapy among pediatric patients, both modalities led to declines in processing speed, but proton therapy, unlike photon therapy, was not associated with significant declines in global IQ or working memory [76]. More specific neurocognitive outcomes of proton radiotherapy are discussed below.

##### Memory Impairment

Radiation to the brain is associated with neurological complications, such as impairments in memory function. This cognitive decline is due to radiation damage and impairment of neurogenesis at the hippocampus [77]. Yang et al. reported the incidence of grade 1 memory impairment in a patient out of 20 treated with hypofractionated proton CSI for leptomeningeal metastases [68]. Moreover, the results of a study conducted by the UK National Health Service for the safety of PBT for CNS, base of skull, spinal, and paraspinal tumors containing a total of 702 patients report the case of one patient who experienced grade 3 memory impairment by the end of treatment [18]. A prospective trial comparing the effects of PBT to that of intensity-modulated radiotherapy for 67 patients diagnosed with glioblastoma reported the incidence of 3 grade 1 cases of memory impairment in the PBT arm of the study. In the IMRT arm, two cases of grade 1 memory impairment were recorded [66].

##### Dysphasia

Dysphasia is a language disorder in which lesions in the dominant hemisphere or brain damage leads to the inability to produce or interpret speech [66]. The incidence of dysphasia has been linked to side effects of conventional radiotherapy [78]. Of the 67 evaluable patients in a prospective trial assessing the outcomes of proton radiotherapy versus intensity-modulated radiotherapy for newly diagnosed glioblastoma, 1 patient was reported to have experienced dysphasia in the PBT arm, and three patients experienced grade 1 dysphasia in the IMRT arm [66].

##### Somnolence Syndrome

Somnolence syndrome usually occurs several weeks after cranial irradiation, presenting as excessive sleep, lethargy, and anorexia [79]. This syndrome is reported as a side effect of conventional radiotherapy; however, there are not enough data to determine the incidences of somnolence syndrome after proton radiotherapy. In a prospective trial containing 20 evaluable patients treated with PBT for CNS tumors, 1 of them developed acute-onset somnolence syndrome that required medical intervention [33]. In conventional photon modalities, somnolence syndrome is well reported as an early delayed post-irradiation side effect, so it is to be expected although underreported as well in proton treatments [80].

#### 3.2.10. Myelitis

Radiation myelitis is a rare and serious complication resulting from radiation exposure to the spinal cord. The pathophysiology behind radiation myelitis is thought to be due to endothelial and oligodendrocyte damage following radiation [81]. Even with photon modalities of radiation therapy, cases have been rare but are still present in the literature [82]. The link between PBT and myelitis is underreported. Gordon et al. reported 1 case of grade 3 myelitis in a single-center study describing the results of PBT in 31 patients with chordomas and chondrosarcomas. This patient had a spinal metal construction that caused range uncertainties in PBT planning due to image artifacts and density [28]. Moreover, Gaito et al. evaluated the outcomes of 702 PBT patients treated in the UK Proton Overseas Programme. One craniopharyngioma patient reportedly developed grade 4 myelitis [18]. Risk factors for the development of radiation myelitis include prior radiation, concurrent chemotherapy, and exceeding dose constraints [82]. All mentioned neurological toxicities are summarized in Table 1. 

### 3.3. Pulmonary Toxicities

#### 3.3.1. Pneumonitis and Fibrosis

Radiation leads to the loss of the alveolar barrier function due to the destruction of endothelial and epithelial cells. This induces a cycle of increased inflammation, increased vascular permeability, and cytokine release early on known as radiation-induced pneumonitis [83,84]. Conventional radiation therapy has been long linked to pneumonitis; however, proton-induced pneumonitis remains relatively underreported [85,86]. In a prospective trial aiming to assess outcomes of 25 patients treated with hypofractionated PBT for thoracic malignancies, 1 patient treated for non-small-cell lung cancer (NSCLC) developed a possible case of grade 3 pneumonitis and pneumonia. Gomez et al. relate the incidence of grade 2+ radiation pneumonitis to a higher mean lung dose (13 Gy vs. 11.3 Gy for the entire group) and higher gross tumor volume (125 cm^3^ versus 53.0 cm^3^ in the entire group). Also, other trials using different chemoradiotherapy modalities have been reported to lead to similar results [87]. Hoppe et al. report the safety outcomes from a multicenter trial evaluating hypofractionated PBT with concurrent chemotherapy for NSCLC in 18 patients. A 79-year-old female was hospitalized 5 months after proton radiation treatment due to the development of grade 4 radiation-induced pneumonitis [88]. In another prospective trial, patient outcomes and toxicities were evaluated for the treatment of stage II-IIIB NSCLC after intensity-modulated proton therapy (IMPT) or IMRT with simultaneous integrated boost (SIB) and concurrent chemotherapy. Two of the fifteen total patients treated with IMPT SIB developed grade 3+ pneumonitis. One of them developed grade 3 pneumonitis 3 months post-treatment, and the other developed grade 5 pneumonitis 2 months post-treatment. It is worth mentioning that the patient who experienced a grade 5 pulmonary event had significant comorbidities. This patient was found to have lung infiltration mainly outside the radiation fields; however, death was still considered treatment-related [89]. Moreover, a prospective randomized trial comparing radiation pneumonitis incidence after PSPT or IMRT for advanced NSCLC found that 10.5% of the total 57 PSPT-treated patients suffered from grade 3+ radiation-induced pneumonitis. Notably, despite patients receiving PSPT having larger tumor volumes (320.7 cm^3^ vs. 257.7 cm^3^), the mean lung dose was similar between proton and IMRT cohorts (16.1 Gy vs. 16.6 Gy). In this study, the rate of radiation pneumonitis was numerically but not significantly higher for PSPT (10.5% vs. 6.5%, *p* = 0.537), although 2 deaths from pneumonitis occurred in the IMRT arm whereas no grade 4–5 pneumonitis events occurred in the PSPT arm. Notably, pneumonitis was seen in 6 of the first 30 PSPT-treated patients but in none of the final 27 PSPT enrolled developed pneumonitis, demonstrating a notable learning curve when delivering PSPT to moving targets like lung cancer [90]. A phase 2 trial evaluating the long-term survival and quality of life of operable stage 1 NSCLC reports the incidence of four cases of grade 2 and one case of grade 3 radiation pneumonitis [91]. Another phase 2 trial evaluated the outcomes following PBT reirradiation for thoracic occurrences of NSCLC with consolidation immunotherapy. Of the evaluable 22 patients, 4 patients had ≥2 pneumonitis, of which 1 received no consolidate pembrolizumab. One case of grade 3 pneumonitis was also reported. In addition, one patient died from sudden-onset grade 5 hemoptysis leading to cardiopulmonary arrest 46.8 months after reirradiation. Moreover, this is expected, as Yegya-Raman et al. explain how dose constraints were not respected since the latest dose constraints were not available at the time when the trial was conducted, and not much time had passed from the prior irradiation [92]. In a prospective trial, Nguyen et al. reported 2 cases of grade 3 radiation-induced pneumonitis among 134 patients diagnosed with stage II-III inoperable NSCLC receiving PSPT with weekly chemotherapy [93]. Moreover, 35 patients with early-stage NSCLC were assessed for dose-escalated PBT treatment toxicities, of which 11.4% reported grade 2 radiation pneumonitis and 2.9% reported grade 3 radiation pneumonitis after proton radiation treatment [94]. A meta-analysis comparing 72 photon studies to 9 proton studies for early-stage NSCLC demonstrated that 0.9% of patients receiving PSPT developed grade ≥ 3 pneumonitis, notably lower than the 3.4% of patients receiving photon therapy [95]. Additional literature reports low-grade expected radiation pneumonitis cases at or modestly below the rates seen with photon therapy [66,96,97,98,99,100,101,102]. Also, similarly to what is observed with photons, the risk of symptomatic pneumonitis is essentially associated with dose to lung. Photon dose constraints of mean lung dose <13.5 Gy and lung V20 < 33.5% minimize risks of pneumonitis in a proton setting as well [103].

Months after radiation exposure to lung tissue, fibrosis is present. It is due to the presence of hyperplastic pneumocytes, increased myofibroblasts, and lower pulmonary volume. This results from the collapsing of alveolar space after extensive collagen deposition in the pulmonary interstitium and alveoli [83]. Nagano et al. described the case of a 76-year-old man with a relapse of squamous cell carcinoma of the lung in the left lobe after a right lobe lobectomy 1 year prior. After the patient received PBT for the relapse, the initial pulmonary fibrosis was exacerbated. These authors concluded that exacerbation of idiopathic pulmonary fibrosis may occur after PBT [104]. Moreover, a 47-year-old man with stage III lung adenocarcinoma developed radiation pneumonitis 7 weeks after IMPT. The patient was later treated with steroids, which resolved his pneumonitis, but he was left with chronic pulmonary fibrosis [105]. Cases of radiation pneumonitis can progress to chronic pulmonary fibrosis, so higher mean lung doses, irradiated volume, and dose per fraction ultimately increase the incidence of radiation-induced lung injury and fibrosis [106].

#### 3.3.2. Pleural Effusion

Pleural effusions occur when an abnormally large amount of fluid collects between the parietal and visceral pleura [107]. The exact pathophysiology behind the cause of radiation-induced pleural effusion is yet to be studied. A study evaluating 55 irradiated patients with pleural effusions found no distinctive cytologic change [108]. Concurrent chemotherapy, mean lung dose, and total volume irradiated are risk factors for the development of pleural effusion in the setting of thoracic irradiation [109]. Conventional-radiation-therapy-induced pleural effusions are well reported in the literature; however, reports of particle-radiation-induced pleural effusions are scarce [110]. A case study on a 47-year-old female with a 10-year locoregional recurrence of breast cancer treated with PBT reported the occurrence of a pleural effusion with clinical burden of dyspnea 19 months after treatment [111]. Moreover, in a randomized trial aiming to compare outcome differences in treating locally advanced esophageal cancer patients with PBT or IMRT, 13 out of 46 patients in the PBT arm were reported to have developed pleural effusions. Of these 13 patients, one required medical intervention, and another required surgical intervention. This is in contrast to the IMRT arm, where 24 out of 61 patients developed pleural effusions, 3 of whom required medical intervention [100]. Hoppe et al. report the case of a low-grade pleural effusion in a patient enrolled in a trial assessing the safety of hypofractionated PBT with concurrent chemotherapy for locally advanced NSCLC in 18 patients [88]. In a phase 2 study evaluating the outcomes of PBT and concurrent chemotherapy of 64 patients for unresectable stage III NSCLC, Chang et al. reported 3 cases of acute grade 1 or 2 pleural effusions, 2 cases of grade 3 late-onset pleural effusions, and 21 cases of late-onset grade 1 or 2 pleural effusions [96].

#### 3.3.3. Bronchial Toxicities

Bronchial toxicities, such as fistulas and strictures, are to be expected when undergoing radiation treatments to the thoracic cavity [112]. Bronchial stricture or stenosis is the narrowing of the airway in the lung due to injury [113]. A bronchial fistula is an abnormal tract between the main stem, lobar, or segmental bronchus of the lung and the pleural space [114]. In a study aiming to evaluate the use of PBT and concurrent chemotherapy for the treatment of unresectable stage III NSCLC, two patients developed a grade 2 bronchial stricture, and one patient developed a grade 4 bronchial fistula [96]. All mentioned pulmonary toxicities are summarized in Table 2.

### 3.4. Cardiac Toxicities

Cardiac dysfunction and damage may result from the destruction of myocytes or agents that affect ventricular contraction [115]. Radiation-induced heart disease is a major concern with patients undergoing radiation treatments to the thoracic area. Conventional photon therapy has been reported extensively in the literature to induce cardiotoxicities; however, proton-therapy-induced adverse cardiac toxicities still require more follow-up [116]. In a comparative study of two independent cohorts treated with either PSPT (86 patients) or IMPT (53 patients) in NSCLC patients, 5% of the PSPT group experienced grade 3 cardiac toxicity, 2% experienced grade 4 cardiac toxicities, and 5% experienced grade 5 cardiac toxicities. When the authors compared the results of the trial to anteceding retrospective and dosimetric studies that expect improvements in clinical outcomes, no reduction in cardiac toxicity was seen, although they noted significantly lower rates of cardiac toxicities among patients treated with IMPT relative to older passive scattering proton therapy [117]. Also, PBT has been linked to cases of heart palpitations and tachycardia as possible post-treatment complications [118]. However, it is worth mentioning that several studies have reported a decreased radiation dose to the heart when proton radiation is used in comparison to photon radiation. This explains the scarcity of cardiac toxicities in the literature. Longer-term research is required to assess the occurrence of late cardiac toxicities which are the main sequelae of radiation injury to the heart [119,120,121]. Table 3 summarizes the cardiac toxicities that will be discussed. 

#### 3.4.1. Pericarditis

The most common type of pericardial disease is known as pericarditis, which results from the inflammation of pericardial tissue. This inflammation results from the irritant itself or due to the release of necrotic debris after radiation [122]. Conventional radiation treatments have been extensively linked to pericarditis; however, proton radiation treatments are underreported in the literature [123]. Lin reported pericarditis in a 47-year-old female patient treated for 10-year locoregional recurrent luminal A breast cancer from November to December 2016 [111]. Laughlin et al. report the occurrence of late aggressive sequalae following an intense PBT course for unresectable mediastinal and pericardial spindle cell sarcoma in a 17-year-old male. Five years following the treatment of chemoradiotherapy that consisted of an initial dose of 45 GyRBE at 1.8 GyRBE per daily fraction, followed by two sequential boosts for a total dose of 64.8 GyRBE at 1.8 GyRBE per daily fraction, the patient suffers from restrictive pericarditis and heart failure. The authors report therapeutic success; however, the late toxicities are still to be addressed [124].

#### 3.4.2. Pericardial Effusion

The pericardium is a fibroelastic sac that contains the heart and proximal great vessels. A pericardial effusion occurs when the volume of serous fluid in the pericardium exceeds normal amounts. Moreover, radiation is a known cause of acute- and late-onset pericardial effusions [125]. In a randomized trial aiming to assess the toxicity advantages of PBT compared to that of IMRT for locally advanced esophageal cancer, 2 cases of asymptomatic pericardial effusion were reported out of 46 from the PBT arm, and 6 cases of asymptomatic pericardial effusion out of 61 in the IMRT [100]. Moreover, in a prospective trial conducted between July 2009 and April 2014 where 201 patients were treated with PBT or IMRT as well as chemotherapy for advanced NSCLC, 86 patients developed grade 2 or 3 pericardial effusion [126]. A case study also reported a case of pericardial effusion with grade 4 cardiac tamponade following the initiation of immunotherapy that was started approximately 8 months following the completion of thoracic PBT [127].

#### 3.4.3. Atrial Fibrillation

Atrial fibrillation (AF) occurs when the atria contract rapidly and non-functionally due to the rapid and irregular depolarization of the atria [128]. Moreover, AF can develop due to pericarditis or cardiomyopathy after radiation treatments [129]. However, proton-radiation-induced AF is not commonly reported in the literature. Lin et al. report a case of atrial fibrillation after PBT (out of 46 patients) in a trial aiming to compare outcomes of treatment with PBT or IMRT for locally advanced esophageal cancer [100]. In a prospective study assessing the long-term outcomes of early-stage NSCLC treated with dose-escalated PBT in 35 patients, Chang et al. reported grade 2 AF in 2 patients, 1 of which experienced grade 2 AF 4 months after radiation treatment and the other 8 months after treatment [94].

**Table 3:** Summary of cardiac toxicities reported following proton radiation.

Paper	Type of PBT	Toxicity Grade	Prescribed Dose	Dose to Structure/OARs	QUANTEC Fractionated Photon 3DCRT Dose Constraints
Pericarditis					
Y. L. Lin [111]	PBS (single-field breast) ^†^	Acute	64.40 GyRBE (total) 56 GyRBE in 28 fr (upper mediastinal, retrosternal lymphatics and entire sternum)	Spinal cord: 0.06 Gy (mean) Right brachial plexus: 43.70 Gy (mean) Left lung: 7.38 Gy (mean) Right lung: 14.79 Gy (mean) Esophagus: 22.89 Gy (mean) Heart: 13.06 Gy (mean) Heart volume receiving ≥30 Gy: 20%	Spinal cord: 50 Gy (max) Lung: 7 Gy (mean) Esophagus: <34 Gy (mean) Heart: <26 Gy (mean)
Laughlin et al. [124]	IMPT	Unspecified (chronic restrictive pericarditis)	64.8 GyRBE (total)	NA	Spinal cord: 50 Gy (max) Lung: 7 Gy (mean) Esophagus: <34 Gy (mean) Heart: <26 Gy (mean)
Pericardial effusion					
Lin et al. [100]	PSPT or PBS *	Asymptomatic	50.4 Gy/28 fr	Lung: 4.8 Gy (mean) Heart: 11.3 Gy (mean) Liver: 2.4 Gy (mean) Spinal cord: 38.3 Gy (mean)	Lung: 7 Gy (mean) Heart: <26 Gy (mean) Liver: <30–32 Gy (mean) Spinal cord: 50 Gy (max)
Ning et al. [126]	PSPT or IMRT *	Grades 2, 3	74 Gy (median) in 2 Gy/fr	Heart: 12.2 Gy (median mean)	Heart: <26 Gy (mean)
McCusker MG et al. [127]	PBS	Unspecified	54 Gy (total dose)	Pleura: 45.0 Gy in 1.8 Gy fractions Right hemithorax: 4.0 Gy sequential boost in 2.0 Gy fractions	Right lung V40 ≤ 67% Left lung mean ≤ 2 Gy Left lung V20 ≤ 5%, Total lungs minus GTV ≤ 20 Gy
Atrial fibrillation					
Lin et al. [100]	PSPT or PBS *	NA	50.4 Gy/28 fr	Lung: 4.8 Gy (mean) Heart: 11.3 Gy (mean) Liver: 2.4 Gy (mean) Spinal cord: 38.3 Gy (mean)	Lung: 7 Gy (mean) Heart: <26 Gy (mean) Liver: <30–32 Gy (mean) Spinal cord: 50 Gy (max)
Chang et al. [94]	Dose-escalated hypofractionated PSPT	Grade 2: 2 patients	87.5 GyRBE in 35 fr (2.5 GyRBE/fr)	Dose limit: Bronchial tree and large blood vessels: 87.5 GyRBE < 10 cm^3^ Heart: 70 Gy < 10%	Heart: <26 Gy (mean)

IMRT: intensity-modulated radiation therapy; IMPT: intensity-modulated proton therapy PBT: proton beam therapy; PSPT: passively scattered proton therapy; PBS: pencil beam scanning; * treatment may include chemotherapy; ^†^ reirradiation.

### 3.5. Gastrointestinal Toxicities

Radiation-induced tracheoesophageal fistulas and esophageal stenosis are among the gastrointestinal (GI) toxicities that have been underreported to date with PBT treatment. Tracheoesophageal fistulas refer to the presence of a congenital or acquired passageway linking the trachea to the esophagus [130]. A phase I trial treating 25 patients diagnosed with NSCLC with PBT reported the death of a patient 7 weeks after the development of tracheoesophageal fistula, 9 months post-PBT, and 1 month after being put on bevacizumab for recurrent disease [87]. In another prospective study evaluating toxicity outcomes in 17 patients with thoracic esophageal carcinoma receiving concurrent proton and chemotherapy, 1 patient developed a late grade 5 tracheoesophageal fistula with hemorrhage 7 months after treatment. In the same study, one patient developed late grade 3 esophageal stenosis, and another developed grade 3 pyloric stenosis [131]. Late grade 3 esophageal stenosis has also been reported in one patient in a prospective trial on the reirradiation of 14 patients with PBT and in 2 patients in another retrospective study, in which 47 patients received concurrent PBT and chemotherapy for esophageal cancers. In the latter study, one patient also developed a grade 3 esophageal fistula [132,133]. Similarly, a separate reirradiation study for esophageal cancer in 17 patients reported 1 patient with grade 4 tracheal stenosis requiring permanent tracheostomy and 1 patient developing a tracheoesophageal fistula requiring surgical intervention [134]. One case of grade 4 esophageal stricture has been observed in a study looking at outcomes after concurrent proton and chemotherapy in 134 patients with NSCLC [93].

Other GI toxicities include esophagitis, pharyngitis, and colitis, which can be defined as inflammation or damage to the esophageal, pharyngeal, or colon mucosa, respectively [135,136,137]. In a phase 2 study on 64 NSCLC patients treated with concurrent proton and chemotherapy, acute grade 3 esophagitis was reported in 8% of patients, and late grade 4 esophagitis in 2% of patients [96]. Grade 3 esophagitis was also observed in 10.6% and 4% of patients in two previously mentioned studies [93,133]. In a case report, acute grade 2 pharyngitis was seen in a patient undergoing PBT for a head and neck recurrent desmoid tumor [138]. In a phase 1–2 trial, 1 patient out of 35 treated in the phase 2 portion developed grade 3 colitis when treated with concurrent PBT and chemotherapy for pancreatic ductal adenocarcinomas [139]. A phase 2 trial assessing outcomes and toxicities following PBT reirradiation reports the occurrence of a grade 5 aorto-esophageal fistula 6.9 months following treatment. The authors state that this was expected due to the unfollowed dose constraints and the short time between the trial and prior irradiation [92]. Another phase 2 study reports the occurrence of a single case out of 94 patients of gastritis following PBT for craniopharyngiomas in pediatric and adolescent patients [140].

Rectal bleeding has also been associated with PBT. In a large prospective study, grade 3 rectal bleeding occurred in 0.9% of patients following PBT for prostate cancer [141]. Similarly, late grade 3 rectal bleeding was reported in 2 patients in another prospective trial evaluating outcomes in 423 patients post-PBT, and in 7 out of 1327 patients in a study looking at outcomes after image-guided proton therapy, both for prostate cancers [142,143]. In a planning study estimating late GI toxicities using multivariable normal tissue complication probability (NTCP) models in patients with prostate cancer treated with either IMPT or IMRT, the average difference in NTCP for grade 2 rectal bleeding between both treatment arms was 12.17% in favor of the IMPT plans [144].

Moreover, elevations in biliary enzymes in hepatocellular carcinoma (HCC) patients following PBT treatment have been reported. In one study, temporarily elevated (grade 2 or 3) aminotransferase, alanine aminotransferase, gamma-glutamyl transpeptidase, and total bilirubin were observed as markers of liver damage in 1.2%, 0.4%, 1.2%, and 3.5% of patients, respectively, that initially had normal pretreatment levels [145]. A multi-institutional cohort study looking at HCC patients treated with either proton or photon therapy reported a significantly higher risk of radiation-induced liver disease in the photon arm versus the proton arm (36% vs. 11.8%, respectively; *p* = 0.004) [146]. In a phase 2 study evaluating the safety and efficacy of PBT in 30 patients with HCC, 4 patients died due to radiation-induced hepatic insufficiency, and grade 3 elevated total bilirubin and transaminases were reported in 1 and 5 patients, respectively, within 90 days from the initiation of treatment [147]. A randomized clinical trial aiming to compare toxicity following transarterial chemoembolization or PBT for hepatocellular carcinoma reported the occurrence of a case of grade 4 and 2 cases of grade 2 hyperbilirubinemia out of the 36 PBT-treated patients [148]. In a retrospective study evaluating the outcomes of 27 HCC patients treated with PBT, 2 patients developed acute hepatic failure (grade 4 hyperbilirubinemia and grade 4 hepatic coma) during their second course of PBT [149].

Finally, a retrospective study comparing the frequency of GI toxicity between 1544 patients treated with IMRT and 772 patients treated with PBT for prostate cancer found no significant differences between both radiation options. The rate of GI toxicity at 6, 12, and 24 months was 3.5% vs. 2.5% (*p* = 0.18), 9.5% vs. 10.2% (*p* = 0.18), and 20.5% vs. 23.4% (*p* = 0.11) for patients undergoing treatment with IMRT vs. PBT, respectively [150]. Discussed gastrointestinal toxicities are summarized in Table 4. 

### 3.6. Hematological Toxicities

Hematological toxicities that have been described in the literature to be associated with radiation therapy include leukopenia, lymphopenia, neutropenia, thrombocytopenia, and anemia. A recent retrospective study assessing acute hematological toxicities following PBT in 20 pediatric patients with brain embryonal tumors that had received prior chemotherapy reported a decrease in leukocyte and neutrophil counts right at the initiation of treatment, with grade 3–4 leukopenia and neutropenia in 35% and 70% of patients, respectively, occurring halfway through treatment [151]. A phase 1 study assessing the safety and treatment outcomes of hypofractionated PBT with adjuvant chemotherapy for the treatment of pancreatic ductal adenocarcinoma reports the incidence of grade 3 prolonged neutropenia in one out of six patients [152]. Another study looking at the incidence of grade 4 lymphopenia in 144 patients diagnosed with esophageal cancer reported a significantly lower incidence in patients undergoing treatment with proton radiation over photon radiation (22% vs. 56%; *p* < 0.01) [153]. Yegya-Raman et al. report the occurrences of several hematological toxicities following reirradiation of thoracic recurrences of NSCLC with PBT. Out of the 22 evaluable patients, 2 had grade 2 neutropenia and 1 had grade 3 neutropenia. Two patients also developed grade 2 lymphopenia, and another suffered from grade 3 lymphopenia following PBT. In addition, a patient developed grade 1 anemia after PBT reirradiation with concurrent immunotherapy [92]. Moreover, a retrospective study evaluating acute toxicities in 50 patients treated with proton craniospinal irradiation, of which 80% had also undergone chemotherapy, reported grade 3 leukopenia in 9% of patients, grade 2 anemia in 15% of patients, and grade 3–4 thrombocytopenia in 4% of patients [154]. In a phase II study evaluating outcomes for concurrent proton radiation and chemotherapy in 15 patients with NSCLC, grade 3 leukocytopenia was reported in 10 patients, grade 3 and 4 neutropenia was found in 3 and 4 patients, respectively, and grade 3 thrombocytopenia was observed in 1 patient [102]. In another phase I prospective trial, of 24 patients with leptomeningeal metastases receiving proton craniospinal irradiation, 3 patients developed grade 3 leukopenia, 2 patients developed grade 4 lymphopenia and thrombocytopenia, and 1 patient developed grade 3 anemia [68]. Moreover, a multi-institutional retrospective study evaluating hematological toxicities in 97 patients with medulloblastoma undergoing treatment with photon or proton therapy and with or without concurrent chemotherapy reported grade 4 leukopenia (1% vs. 0%; *p* = 0.044), grade 4 lymphopenia (67.6% vs. 16.9%; *p* = 0.0001), and grade 3 anemia (8.1% vs. 0.0%; 0.011) in the photon vs. proton arms, respectively [155]. The aforementioned hematological toxicities are summarized in Table 5. 

### 3.7. Urological Toxicities

#### 3.7.1. Genitourinary Toxicities

Several genitourinary (GU) toxicities have been reported following PBT. In a recent study looking to assess acute GU toxicities in 227 patients diagnosed with prostate cancer who were treated with either normo-fractionated proton therapy (NFPT) (*n* = 107) or moderately hypofractionated proton therapy (MHPT) (*n* = 120), the most common GU toxicity was grade 1 (29% vs. 28%; *p* = 0.02) and grade 2 (36% vs. 23%; *p* = 0.02) urinary retention in the NFPT and MHPT groups, respectively. The least experienced toxicity was grade 1 (0.9% vs. 0.8%; *p* = 0.28) and grade 2 (0.9% vs. 0%; *p* = 0.28) hematuria in the NFPT and MHPT groups, respectively. Among the other GU toxicities reported were urinary frequency, urgency or incontinence, and urinary tract pain [156]. Moreover, a retrospective study assessed late toxicities in 93 prostate cancer patients undergoing PBT and reported one case of grade 3 hematuria and non-infectious cystitis, as well as one case of grade 2 urinary frequency [157]. In another retrospective study evaluating treatment outcomes following image-guided proton therapy for 1327 patients with prostate cancers, 5.4% of patients experienced grade 3+ GU toxicities, including both acute and late grade 3 bladder irritation, hematuria, and urinary obstruction, with 1 case of grade 4 hematuria [142]. Bowel perforation, which may have occurred due to the tumor’s proximity to the bowel structures, is another toxicity that has been reported as grade 3 in 1 pediatric patient with Ewing sarcoma of the pelvis receiving both proton therapy and surgery, out of the 35 patients treated [158]. In a previously mentioned retrospective study, the rate of GU toxicity was also compared between 1544 patients treated with IMRT and 772 patients treated with PBT for prostate cancer. Similarly to the results found with GI toxicities, no significant differences were reported in terms of the frequency of GU toxicities in both groups. The rate of GU toxicity at 6, 12, and 24 months was 6.8% vs. 5.7% (*p* = 0.30), 14.3% vs. 12.2% (*p* = 0.13), and 28.2% vs. 25.8% (*p* = 0.21) for patients undergoing treatment with IMRT vs. PBT, respectively [150].

#### 3.7.2. Reproductive-Related Toxicities

As for reproductive toxicities, a case study on a 65-year-old male with prostate and rectal cancers reported the loss of erectile function following treatment with PBT, chemotherapy, and resection [159]. A retrospective study looking to assess erectile function and urinary incontinence in 262 patients with prostate cancer following PBT found that 4% of patients reported severe erectile dysfunction 2 years post-treatment [160]. Recent results from a prospective phase II trial in which 25 patients with cervical and endometrial malignancies were treated with PBT alone or in combination with chemotherapy reported the incidence of urinary incontinence, urinary frequency, nocturia, and vaginal bleeding [161]. Grade 1–2 vaginal mucosal toxicity, which involved telangiectasias, shortening of the vagina on exam, and fibrosis, was reported in a case study on a patient undergoing reirradiation with PBT for vaginal carcinoma [162]. Similarly, another case report on a patient undergoing reirradiation with PBT for vaginal squamous cell carcinoma revealed late grade 1 vaginal dryness and atrophy [163]. Grade 2 premature ovarian deficiency has also been reported in 1 patient out of 35 undergoing PBT for Ewing sarcoma of the pelvis [158]. Urological toxicities are summarized in Table 6.

### 3.8. Osteological Toxicities

Osteoradionecrosis refers to the necrosis of bone that may arise following high doses of radiation to head and neck cancers and lasts for 3 months or longer without healing spontaneously [164]. A recent case series of 122 patients reported a prevalence of 10.6% for osteoradionecrosis following PBT, inclusive of patients with oral and oropharyngeal cancers who were asymptomatic with radiographic evidence of osteoradionecrosis, along with 9 patients with grade 2 and 1 patient with grade 3 osteoradionecrosis [165]. A retrospective study comparing the incidence of osteoradionecrosis with radiation dose in patients with oropharyngeal malignancies undergoing treatment with IMRT (*n* = 534) vs. IMPT (*n* = 50) identified a higher incidence of osteoradionecrosis in the IMRT group (7.7%), which was associated with higher radiation doses, compared to the lower dose and incidence in the IMPT group (2.2%) [166]. Only 1 patient experienced grade 1 osteonecrosis in the IMPT group, whereas grade 4, grade 3, grade 2, and grade 1 osteoradionecrosis were reported in 12, 5, 1, and 23 patients in the IMRT group [166]. Other osteological adverse events have been described in a previously mentioned study on PBT in 35 patients with pelvic Ewing sarcoma, in which 1 patient developed grade 2 unequal limb length and another suffered a grade 3 hip dislocation [158]. Grade 2 rib fractures were also reported in 1 patient out of 35 on a phase I/II prospective study and in 4 patients out of 111 in another study looking at early-stage NSCLC treated with PBT [94,167]. Table 7 provides a summary of the osteological toxicities discussed.

### 3.9. Oral-Health-Related Toxicities

Abnormalities in dental development due to radiation have been commonly underreported. A recent case report on a pediatric patient diagnosed with an embryonal rhabdomyosarcoma revealed several late dental toxicities 3.5 years after receiving a combination of PBT and chemotherapy, which included microdontia, hypodontia, hypomineralization of the first molars and central incisors, abruption of root development, and partial eruption and lack of eruption of teeth [168]. Another oral late toxicity reported after radiation is xerostomia, described as the subjective sensation of a dry mouth, which may also include a decrease in salivary flow [169]. In a study looking at the quality-of-life outcomes related to xerostomia after treatment with IMPT in 69 patients with oropharyngeal malignancies, patients continued to report xerostomia-related quality-of-life impairment at above baseline levels 2 years after treatment [170]. Moreover, a retrospective study comparing rates of xerostomia after treatment with IMRT (*n* = 249) versus IMPT (*n* = 103) in patients with oropharyngeal cancer reported no significant differences between both treatment arms for the incidence of moderate–severe xerostomia at 18 months post-treatment. However, patients receiving IMPT had a decreased incidence of moderate–severe xerostomia 18–24 months (6% vs. 20%; *p* = 0.025) and 24–36 months (6% vs. 20%; *p* = 0.01) after treatment compared to the IMRT group [171]. Other commonly reported toxicities include dysgeusia (taste impairment), dysphagia (difficulty swallowing), and oral mucositis. In a retrospective study evaluating acute toxicities in 105 patients with major salivary gland cancers following PBT, grade 2 oral mucositis, dysphasia, xerostomia, and dysgeusia were reported in 10.5%, 8.6%, 7.6%, and 4.8% of patients, respectively. Grade 3 oral mucositis and dysphagia were also found in 2.9% and 1.9% of patients, respectively [172]. Oral-health-related toxicities are summarized in Table 8.

### 3.10. Endocrine Toxicities

Endocrinopathies are common side effects of radiation treatments of cancers [173]. Radiation-induced endocrinopathies include growth hormone, thyroid hormone, and gonadal hormone deficiencies. These deficiencies are due to direct endocrine organ damage and/or abnormalities in respective endocrine axes [174]. Proton-radiation-induced endocrinopathies remain relatively underreported with respect to conventional radiation modalities in the literature. In a prospective trial aiming to assess the outcomes of treating benign meningioma patients with two different doses of fractionated proton–photon therapy, several endocrinopathies were recorded. Of the 44 evaluable patients, 16 suffered from grade 2 late-onset endocrine deficits (6 patients in the 55.8 Gy arm and 10 patients in the 63.0 Gy arm), and 1 suffered from grade 4 late-onset endocrine deficits (55.8 Gy arm). As for acute toxicities, one patient suffered from a grade 2 endocrine deficit (63 Gy arm) [61]. In another prospective trial, Ladra et al. evaluate the preliminary results of a phase II trial for treatment with proton radiotherapy in pediatric rhabdomyosarcoma. Of the 57 patients, 3 patients that received radiation doses to the hypothalamus or pituitary suffered from late endocrine dysfunction, including grade 2 growth hormone deficiency in 2 patients and combined grade 2 thyroid and growth hormone deficiency in 1 patient [75]. Moreover, Bishop et al. performed a multi-institutional analysis of toxicities after PBT or IMRT treatment for childhood craniopharyngioma and reported that 16 out of the 21 patients treated with PBT developed late post-treatment endocrinopathies, 7 patients treated with PBT developed late-onset panhypopituitarism after treatment, and 16 patients developed growth hormone deficits, hypothyroidism, adrenal insufficiency, and sex hormone deficiencies. The authors report similar late toxicity outcomes between PBT and IMRT patients [175]. Moreover, the incidence of endocrinopathies was evaluated in an analysis report of a prospective trial for proton-therapy-treated medulloblastoma patients compared to a retrospective trial for photon therapy-treated medulloblastoma patients. Eaton et al. provide a univariate analysis of endocrine dysfunction: of the 40 chemotherapy- and proton-radiation-treated patients; 21 had late-onset growth hormone deficiency, 2 had adrenal insufficiency, 1 had sex hormone deficiency, and 22 patients underwent endocrine replacement therapy. The results show that PBT was associated with a decreased risk of sex hormone deficiency (3% vs. 19%, *p* = 0.025) and requirement for any endocrine replacement therapy (55% vs. 78%, *p* = 0.030). No significant differences were reported in the incidence of growth hormone deficiency (53% vs. 57%) or adrenal insufficiency (5% vs. 8%) [176]. A prospective trial evaluated the toxicities and outcomes of low-grade glioma patients after fractionated PBT. Of 20 patients, 3 developed post-treatment central hypothyroidism, 4 developed new adrenal insufficiency, and 2 male patients developed central hypogonadism. This is expected since all but one patient had received direct irradiation to the hypothalamic–pituitary axis [71].

Indelicato et al. assessed the outcomes of PBT in 179 pediatric ependymoma patients between 2007 and 2017 at the University of Florida. In their study, 13 patients were reported to have experienced late grade 2+ endocrine deficiencies. The authors state that no unexpected events occurred [54]. In another prospective study, pediatric patients were evaluated for outcomes following PBT for CNS germ cell tumors. Of the 20 evaluable patients, 7 developed post-treatment growth hormone deficiency, 1 developed growth hormone deficiency and hypothyroidism, and 1 patient developed hypogonadism attributed to both PBT and chemotherapy. Growth hormone deficiency being the most common radiation-induced endocrine toxicity in this study is expected, since most cases involved irradiation of the hypothalamic–pituitary axis, where deficiencies have been previously reported at doses lower than 18 Gy [33].

The incidence of endocrinopathies is correlated with an increased radiation dose to endocrine organs [177]. Several studies provide evidence of fewer endocrinopathies following PBT when compared to conventional radiation modalities, due to its sparing of endocrine non-target tissues [176,178,179]. Table 9 summarizes the discussed endocrine toxicities.

## 4. Discussion

In this review, commonly reported toxicities (like fatigue, headaches, and skin dermatitis) were not included. Common toxicities are frequently encountered with all radiation modalities and are not specific to proton radiation treatments [180]. All other toxicities mentioned in this review are relatively underreported and/or pose significant clinical risks to radiation patients. Almost all mentioned proton-related toxicities are underreported when compared to conventional radiation toxicities. Hence, less published evidence of their occurrences, when compared to photon RT, exists in the literature, which makes it harder to draw strong conclusions regarding the relationship between the radiation parameters and the incidence and prevalence of proton RT toxicity. This can be attributed to the smaller number of patients getting treated with protons in comparison with conventional photon radiation therapy. Of the estimated half of all cancer patients who eventually receive radiation treatments, only 279,455 patients have received proton radiation therapy worldwide as of December 2021 [3,181]. This low number is related in part to the cost-effectiveness of establishing and maintaining proton therapy centers [6]. Physicians are therefore encouraged to report on the outcomes of proton radiation therapy when possible and to provide a more detailed presentation of cases where all dose volume radiation parameters and used constraints are detailed. This is important for providing a clearer understanding of whether a toxicity is expected or unexpected. Failure to do so serves as a shortcoming in assessing the cause of radiation injury when it comes to proton radiation. Also, uniformity in reporting toxicity outcomes, such as using the latest Common terminology Criteria for Adverse Events (CTCAE), is encouraged to describe and evaluate outcomes of patients treated with proton RT.

In general, expected toxicities following RT are commonly seen and not severe (grade < 3), and usually occur when dose constraints are not respected. However as previously mentioned, due to PBT’s unique properties of dose falloff which limits unnecessary radiation exposure to surrounding organs at risk, radiation-related side effects occur to a far lesser extent with proton RT when compared to photon RT. On the other hand, unexpected toxicities are more commonly encountered with proton RT when compared to photon RT, although not consistently reported in the literature. These unexpected toxicities can happen due to range and RBE uncertainties, which can lead to higher exposure of organs at risk to radiation than calculated. To this point, for example, IMRT is still predominantly used in the treatment of pancreatic cancer worldwide to prevent cases of overshooting to the bowel. These unexpected toxicities are usually serious (grade 3+), and occur despite meeting dose constraints. Out of the reported proton RT toxicities, unexpected toxicities, albeit still relatively rare, include high-grade radiation necrosis and fistulae.

The RBE of a proton is simplified to 1.1; however, this is not essentially true. Variability of RBE related to tissue and cell type might interfere with the dose deposition of proton beams [6]. This is especially seen when PBT is used in heterogeneous tissue interfaces such as the lungs. Physical uncertainties also affect the proper delivery of PBT, where the movement of the tumor leads to inconsistent deposits of radiation along the beam’s path [6]. These variabilities of RBE and motion might lead to higher dose depositions in normal tissues, which could explain cases of unexpected toxicities. Ameliorating dose planning and beam delivery techniques are essential to unlock proton radiation therapy’s full potential. Studying the different RBEs of protons across different cell lines and tissue types will provide valuable information to better plan and treat future PBT patients.

## 5. Conclusions

In general, proton RT toxicity profiles are more favorable in comparison with photon RT due to the Bragg peak effect. However, unexpected toxicities are more likely to happen with proton RT, mostly due to RBE and range uncertainties. The current literature lacks large-scale studies including randomized control trials that can accurately quantify toxicity following proton RT compared to photon RT. Several ongoing trials have the potential to tackle this effectively, such as NRG-GI003, NRG-GI006, NCT20603341, NCT01993810, and others. A more comprehensive large-scale reporting of PBT toxicities is needed to further characterize the toxicity profiles associated with proton RT, including unexpected and underreported toxicities. One consideration would be to use a different set of dose constraints, stricter and lower, when planning proton treatments and not duplicate the same constraints used for photon radiation planning. This is especially relevant when treating targets close to critical organs at risk where dose overshooting can lead to drastic complications, such as the cord and luminal GI organs, and in patients with prior courses of RT to the same region.

## Figures and Tables

**Table 1:** Summary of neurological toxicities reported following proton radiation.

Paper	Type of PBT	Toxicity Grade	Prescribed Dose	Dose to Structure/OARs	QUANTEC Fractionated Photon 3DCRT Dose Constraints
Radiation necrosis					
Galle et al. [16]	PSPT ^†^	Grade 1: 1 patient Unspecified: 1 patient (required surgical intervention)	89.4 Gy (cumulative dose) 118 Gy (cumulative dose)	NA	
Gaito et al. [18]	USPT or PSPT	Grade 5: 2 patients	54 GyRBE (median)	NA	
Gordon et al. [19]	PBS ^†^	Unspecified	60 Gy with 2.0 Gy/fr	D95 = 56.3 GyRBE Left temporal lobe: 56.5 GyRBE (max dose) Brainstem: 16.1 GyRBE (max dose) Chiasma: 29.8 GyRBE (max dose) Right optical nerve: 7.8 GyRBE (max dose) Hypophysis: 3.0 GyRBE (mean) Midbrain: 1.0 GyRBE (mean)	Brain: <60 Gy (max) Brainstem: <54 Gy (max) Optic chiasm: <55 Gy
Bronk et al. [20]	PSPT	NA	60 GyRBE/30 fr	D95: <60 Gy RBE (in 6 of 10 lesions)	
Gunther et al. [21]	PSPT (CSI)	Grade 4: 1 patient	30 Gy (patient treatment) 21.8 Gy (median)	NA	
Brainstem toxicity					
Indelicato et al. [24]	PSPT	Grade 2: 7 patients Grade 3: 1 patient Grade 4: 2 patients Grade 5: 1 patient	54 CGE (median)	Brainstem: >50.4 CGE Brainstem V54 CGE: 26%	Brainstem: <54 Gy (max)
Holtzman et al. [26]	PSPT or PBS	Grade 2: 1 patient Grade 3: 1 patient	73.8 GyRBE (median)	Brainstem: 59 GyRBE (Dmax)	Brainstem: <54 Gy (max)
Vogel et al. [27]	PBS *^,†^	NA	54 GyRBE (median)	CSI: 36 GyRBE (median) Brainstem: 55.4 GyRBE (median max dose) Brainstem: 98 GyRBE (cumulative median max dose)	Brain: <60 Gy (max) Brainstem: <54 Gy (max)
Gordon et al. [28]	PBS	Grade 5	70.0 GyRBE (median) in 2 GyRBE/fr	Brainstem: 64.0 GyRBE (Dmax)	Brainstem: <54 Gy (max)
Moyamoya syndrome					
Greenfield et al. [33]	PSPT or PBS *	NA	50.4 GyRBE, Initial: 36.0 GyRBE Boost: 14.4 GyRBE	NA	
Scala et al. [34]	PBS	NA	72.0 GyRBE in 40 fr of 1.8 GyRBE	Tumor bed: 50.4 GyRBE, boost to tumor nodules: 21.6 GyRBE	
Zwagerman et al. [35]	PSPT *	NA	Tumor bed: 52.2 CGE	Hypothalamus: 49.8 CGE (mean), pituitary: 47.8 CGE (mean), Circle of Willis estimated dose: 37–45 CGE	
Reynolds et al. [36]	PSPT *, PSPT (2 patients)	NA	50.4 CGE/28 fr of 1.8 CGE 59.6 CGE/30 fr of 1.9 CGE	NA	
Phthisis bulbi					
Nakagawa et al. [41]	PBS *	NA	45 Gy/25 fr	NA	
Zehetmayer et al. [42]	PSPT	NA	60 CGE	NA	
Phantosomia					
Rosenzweig et al. [50]	PBS-IMPT	Mild to moderate (12 patients)	Median dose: 23.4 Gy/1.8 Gy per fr	NA	
Raghavan et al. [51]	PBS	Severe	NA	Craniospinal axis: 36 CGE Boost thecal sac: 54.0 CGE Boost focal thoracic spine areas: 50.4 CGE	Brain: <60 Gy (max) Spinal cord: 50 Gy (max)
Hearing loss					
Koetsier et al. [53]	PSPT (stereotactic single-dose proton radiosurgery or fractionated stereotactic PBT)	NA	12 GyRBE or 54 GyRBE or 50.4	Cochlea mean dose: 44.2 GyRBE (fractionated arm) 8.9 GyRBE (single dose arm)	Cochlea: ≤45 Gy (mean)
Indelicato et al. [54]	PSPT	NA	59.4 GyRBE (patients >3 years old) 54 GyRBE (patients ≤3 years old)	NA	
Otitis					
Fuji et al. [55]	PSPT	Grade 2	63 GyRBE (median)	Dose constraint: 60 GyRBE (optic nerves and chiasma) <67 GyRBE (D90 max brainstem) <60 GyRBE (max dose to center of brainstem)	Optic nerve/chiasm: <55 Gy Brainstem: <54 Gy (max)
Indelicato et al. [56]	PSPT	2 cases of unspecified grade	73.8 GyRBE (median)	NA	
Brain edema					
Greenfield et al. [33]	PSPT or PBS	4 cases requiring medical intervention	52.2 GyRBE (median)	NA	
Sanford et al. [61]	PSPT or PBS	Grade 4: 1 patient	55.8 GyRBE	NA	
Neuromotor deficits					
Sanford et al. [61]	PSPT or PBS	Grade 4: 1 patient	55.8 GyRBE	NA	
Ataxia					
Upadhyay et al. [64]	PSPT or PBS	Grades 2, 3, 4, 5	54 CGyE (median)	Mean Dmax brainstem: 55.9 Gy	Brainstem: <54 Gy (max)
Saeed et al. [65]	PSPT or PBS *^,†^	Grade 3	46.2 Gy (median)/2.2 Gy per fr (median)	NA	
Brown et al. [66]	PSPT or PBS *	Grade 1	50 Gy and 60 Gy in 30 fr	Whole brain: 65.19 Gy (max) Right lens: 162.81 cGy (max) Left lens: 213.99 cGy (max) Right cochlea: 11.52 Gy (max) Left cochlea: 9.28 Gy (max) Pituitary: 21.35 Gy (max) Right hippocampus: 34.58 Gy (max) Left hippocampus: 38.15 Gy (max)	Brain: <60 Gy (max) Cochlea: ≤45 Gy (mean)
Sanford et al. [61]	PSPT or PBS	Grade 1: 1 patient (55.8 Gy dose) Grade 2: 1 patient (55.8 Gy dose) Grade 1: 1 patient (63.0 Gy dose) Grade 2: 2 patients (63.0 Gy dose)	55.8 GyRBE or 63.0 GyRBE	NA	
Gait disturbance					
Yang et al. [68]	PBS (hypofractionated proton CSI)	Grade 2	30 GyRBE in 10 fr	NA	
Dysarthria					
Shih et al. [71]	PSPT	Grade 1	54 GyRBE/30 fr	NA	
Sanford et al. [61]	PSPT or PBS	Grade 1: 1 patient	55.8 GyRBE	NA	
Cognitive disturbance					
Saeed et al. [65]	PBS or PSPT *^,†^	Grade 3	46.2 Gy (median)/2.2 Gy per fr (median)	NA	
Gaito et al. [18]	USPT or PSPT	Grade 3: 9 patients	54 GyRBE (median)	NA	
Memory impairment					
Yang et al. [68]	PBS (hypofractionated proton CSI)	Grade 1	30 GyRBE in 10 fr	NA	
Gaito et al. [18]	USPT or PSPT	Grade 3	54 GyRBE (median)	NA	
Brown et al. [66]	PBS or PSPT *	Grade 1: 3 patients	Mean whole brain: 19.38 GyRBE, Max: 65.19 GyRBE	Whole brain: 65.19 Gy (max) Right lens: 162.81 cGy (max) Left lens: 213.99 cGy (max) Right cochlea: 11.52 Gy (max) Left cochlea: 9.28 Gy (max) Pituitary: 21.35 Gy (max) Right hippocampus: 34.58 Gy (max) Left hippocampus: 38.15 Gy (max)	Brain: <60 Gy (max) Cochlea: ≤45 Gy (mean)
Dysphasia					
Brown et al. [66]	PSPT or PBS *	Grade 1	Mean whole brain: 19.38 GyRBE, Max: 65.19 GyRBE	Whole brain: 65.19 Gy (max) Right lens: 162.81 cGy (max) Left lens: 213.99 cGy (max) Right cochlea: 11.52 Gy (max) Left cochlea: 9.28 Gy (max) Pituitary: 21.35 Gy (max) Right hippocampus: 34.58 Gy (max) Left hippocampus: 38.15 Gy (max)	Brain: <60 Gy (max) Cochlea: ≤45 Gy (mean)
Somnolence syndrome					
Greenfield et al. [33]	PSPT or PBS	Required medical intervention	52.2 GyRBE (median)	NA	
Myelitis					
Gordon et al. [19]	PBS	Grade 3	70.0 GyRBE (median) in 2 GyRBE/fr	Brainstem: 52.85 GyRBE (Dmax) Chiasma: 45.9 GyRBE (Dmax) Right optical nerve: 43.2 GyRBE (Dmax) Left optical nerve: 44.8 GyRBE (Dmax) Spinal cord: 20.4 GyRBE (Dmax) Right temporal lobe: 18 GyRBE (mean) Left temporal lobe: 16.5 GyRBE (mean) Hypophysis: 52.1 GyRBE (mean)	Brainstem: <54 Gy (max) Optic nerve/chiasm: <55 Gy Spinal cord: 50 Gy (max)
Gaito et al. [18]	USPT or PSPT	Grade 4	54 GyRBE (median)	NA	

PBS: pencil beam scanning; CSI: craniospinal irradiation; IMPT: intensity-modulated proton therapy; PBT: proton beam therapy; PSPT: passively scattered proton therapy; USPT: uniform scanning proton therapy; * treatment may include chemotherapy; ^†^ reirradiation.

**Table 2:** Summary of pulmonary toxicities reported following proton radiation.

Paper	Type of PBT	Toxicity Grade	Prescribed Dose	Dose to Structure/OARs	QUANTEC Fractionated Photon 3DCRT Dose Constraints
Pneumonitis					
Gomez et al. [87]	Hypofractionated PSPT	Grade 3	60 GyRBE in 15 fr of 4 GyRBE	Lung: 13.0 GyRBE (mean)	Lung: 7 Gy (mean)
Hoppe et al. [88]	Hypofractionated PSPT, USPT, or PBS *	Grade 4	60.01 GyRBE in 3.53 GyRBE/fr	Lung: 10 GyRBE (mean)	Lung: 7 Gy (mean)
Jeter et al. [89]	PBS IMPT *	Grades 3 and 5	78 Gy	Lung: 15 Gy (mean)	Lung: 7 Gy (mean)
Liao et al. [90]	PSPT *	Grade 3	74 or 66 GyRBE	Lung: 16.1 GyRBE (mean) Esophagus: 23.6 GyRBE (mean) Heart: 5.9 GyRBE (mean)	Lung: 7 Gy (mean) Esophagus: <34 Gy (mean) Heart: <26 Gy (mean)
Nguyen et al. [93]	PSPT *	Grade 3	74 GyRBE	Spinal cord: Dmax <45 Gy Lung: ≤20 Gy (mean) Esophagus: Dmax ≤ 80 Gy Heart: <26 Gy (mean) Kidney: 20 Gy to <32% of bilateral kidney Liver: <30 Gy (mean)	Spinal cord: 50 Gy (max) Lung: 7 Gy (mean) Esophagus: <34 Gy (mean) Heart: <26 Gy (mean) Kidney: <15–18 Gy (mean) Liver: <30–32 Gy (mean)
Chang et al. [94]	Dose-escalated hypofractionated PSPT	Grade 2: 4 patients, Grade 3: 1 patient	87.5 GyRBE in 35 fr (2.5 GyRBE/fr)	Dose limit: Bronchial tree and large blood vessels: 87.5 GyRBE < 10 cm^3^ Heart: 70 Gy < 10%	Heart: <26 Gy (mean)
Nakajima et al. [91]		Grade 2: 4 patients, Grade 3: 1 patient	66 GyRBE in 10 fractions (peripheral lesions), 72.6 GyRBE in 22 fractions (central lesions)	Lung: 4.6 GyRBE (mean) Heart: 0 GyRBE (mean)	Lung: 7 Gy (mean) Heart: <26 Gy (mean)
Yegya-Raman et al. [92]	PBS or PSPT	Grade ≥ 2: 4, Grade 3: 1	60–70 Gy in fractions	Lung: 6.9 Gy (mean) Heart: 2.5 Gy (mean) Esophagus: 9.7 Gy (mean)	Lung: 7 Gy (mean) Esophagus: <34 Gy (mean) Heart: <26 Gy (mean)
Fibrosis					
Nagano et al. [104]	PSPT	Exacerbation of previous fibrosis	66 GyRBE/10 fr	NA	
Hu et al. [105]	PBS IMPT *	NA	50 GyE/25 fr	Lung: 56.06 GyE (max) Heart: 55 GyE (max) Esophagus: 51.97 GyE (max) Spinal cord: 11.91 GyE (max)	Lung: 7 Gy (mean) Heart: <26 Gy (mean) Esophagus: <34 Gy (mean) Spinal cord: 50 Gy (max)
Pleural Effusion					
Y. L. Lin [111]	PBS (single-field breast) ^†^	NA	64.40 GyRBE (total) 56 GyRBE in 28 fr (upper mediastinal, retrosternal lymphatics and entire sternum)	Spinal cord: 0.06 Gy (mean) Right brachial plexus: 43.70 Gy (mean) Left lung: 7.38 Gy (mean) Right lung: 14.79 Gy (mean) Esophagus: 22.89 Gy (mean) Heart: 13.06 Gy (mean) Heart volume receiving ≥30 Gy: 20%	Spinal cord: 50 Gy (max) Lung: 7 Gy (mean) Esophagus: <34 Gy (mean) Heart: <26 Gy (mean)
Lin et al. [100]	PSPT or PBS *	Asymptomatic: 13 patients Surgical intervention: 1 patient medical intervention: 1 patient	50.4 Gy/28 fr	Lung: 4.8 Gy (mean) Heart: 11.3 Gy (mean) Liver: 2.4 Gy (mean) Spinal cord: 38.3 Gy (mean)	Lung: 7 Gy (mean) Heart: <26 Gy (mean) Liver: <30–32 Gy (mean) Spinal cord: 50 Gy (max)
Hoppe et al. [98]	Hypofractionated PSPT, USPT, or PBS *	Grade 2	60 GyRBE in 2.5 to 3.53 GyRBE/fr	NA	
Chang et al. [96]	PSPT *	Grade 3: 2 patients	74 GyRBE	NA	
Bronchial stricture					
Chang et al. [96]	PSPT *	Grade 2: 2 Patients	74 GyRBE	NA	
Bronchial fistula					
Chang et al. [96]	PSPT *	Grade 4	74 GyRBE	NA	

PBS: pencil beam scanning; IMPT: intensity-modulated proton therapy; PBT: proton beam therapy; PSPT: passively scattered proton therapy; USPT: uniform scanning proton therapy * treatment may include chemotherapy; ^†^ reirradiation.

**Table 4:** Summary of gastrointestinal toxicities reported following proton radiation.

Paper	Type of PBT	Toxicity Grade	Prescribed Dose	Dose to Structure/OARs	QUANTEC Fractionated Photon 3DCRT Dose Constraints
Tracheoesophageal fistula					
Gomez et al. [87]	Hypofractionated PSPT ^†^	Grade 5	52.5 Gy (15 fr of 3.5 Gy)	Esophagus: 21.0 Gy (mean), 55.6 Gy (max)	Esophagus: <34 Gy (mean)
Rutenberg et al. [131]	PSPT *	Grade 5	64.8 GyRBE	45 Gy GyRBE (initial dose to gross disease and elective lymph nodes) Boost range: 5.4–19.8 GyRBE Dose constraints: Lung: 6.5 GyRBE (mean) Heart: 7.8 GyRBE (mean)	Lung: 7 Gy (mean) (mean) Heart: <26 Gy (mean)
DeCesaris CM et al. [134]	PBS IMPT ^‡^	Grade 4	53.4 Gy (median)	Spinal cord: 13.10 Gy (median) Total lung mean: 3.70 Gy Liver mean: 0.30 Gy Heart mean: 0.70 Gy	Lung: 7 Gy (mean) (mean) Heart: <26 Gy (mean) Liver: <30–32 Gy
Esophageal stenosis					
Rutenberg et al. [131]	PSPT *	Grade 3	54 GyRBE	45 Gy GyRBE (initial dose to gross disease and elective lymph nodes) Boost range: 5.4–19.8 GyRBE Dose constraints: Lung: 6.5 GyRBE (mean) Heart: 7.8 GyRBE (mean)	Lung: 7 Gy (mean) (mean) Heart: <26 Gy (mean)
Fernandes et al. [132]	PSPT or PBS *^,‡^	Grade 3	54.0 Gy (median reirradiation dose) 109.8 Gy (median cumulative dose)	NA	
Takada et al. [133]	PSPT *	Grade 3	33–39.6 Gy in 15–18 fr	Dose constraints: Lung: <20 Gy (mean) Lung V20: <35% Heart V40: <40% Liver V30: < 30% Spinal cord: <45 Gy	Lung: 7 Gy (mean) Heart: <26 Gy (mean) Liver: <30–32 Gy (mean) Spinal cord: 50 Gy (max)
Nguyen et al. [93]	PSPT *	Grade 4	74 Gy	Spinal cord: Dmax <45 Gy Lung: ≤20 Gy (mean) Esophagus: Dmax ≤ 80 Gy Heart: <26 Gy (mean) Kidney: 20 Gy to <32% of bilateral kidney Liver: <30 Gy (mean)	Spinal cord: 50 Gy (max) Lung: 7 Gy (mean) Esophagus: <34 Gy (mean) Heart: <26 Gy (mean) Kidney: <15–18 Gy (mean) Liver: <30–32 Gy (mean)
Pyloric stenosis					
Rutenberg et al. [131]	PSPT *	Grade 3	50.4 Gy (median)	45 GyRBE (initial dose to gross disease and elective lymph nodes) Boost range: 5.4–19.8 GyRBE Dose constraints: Lung: 6.5 GyRBE (mean) Heart: 7.8 GyRBE (mean)	Lung: 7 Gy (mean) (mean) Heart: <26 Gy (mean)
Esophageal fistula					
Takada et al. [133]	PSPT *	Grade 3	33–39.6 Gy in 15–18 fr	Esophagus lesion: 75.6 Gy Dose constraints: Lung: <20 Gy (mean) Lung V20: <35% Heart V40: <40% Liver V30: < 30% Spinal cord: <45 Gy	Lung: 7 Gy (mean) Heart: <26 Gy (mean) Liver: <30–32 Gy (mean) Spinal cord: 50 Gy (max)
		Aorto-esophageal fistula			
Yegya-Raman et al. [92]	PBS or PSPT	Grade 5	60–70 Gy in fractions	Lung: 6.9 Gy (mean) Heart: 2.5 Gy (mean) Esophagus: 9.7 Gy (mean)	Lung: 7 Gy (mean) Esophagus: <34 Gy (mean) Heart: <26 Gy (mean)
Esophagitis					
Chang et al. [96]	PSPT *	Grades 3–4	74 Gy	NA	
Nguyen et al. [93]	PSPT *	Grade 3	60–74 Gy	Spinal cord: Dmax <45 Gy Lung: ≤20 Gy (mean) Esophagus: Dmax ≤ 80 Gy Heart: <26 Gy (mean) Kidney: 20 Gy to <32% of bilateral kidney Liver: <30 Gy (mean)	Spinal cord: 50 Gy (max) Lung: 7 Gy (mean) Esophagus: <34 Gy (mean) Heart: <26 Gy (mean) Kidney: <15–18 Gy (mean) Liver: <30–32 Gy (mean)
Takada et al. [133]	PSPT *	Grade 3	33–39.6 Gy in 15–18 fr	Dose constraints: Lung: <20 Gy (mean) Lung V20: <35% Heart V40: <40% Liver V30: < 30% Spinal cord: <45 Gy	Lung: 7 Gy (mean) Heart: <26 Gy (mean) Liver: <30–32 Gy (mean) Spinal cord: 50 Gy (max)
Pharyngitis					
Sawada et al. [138]	PSPT	Grade 2	60 GyRBE in 30 fr	NA	
Colitis					
Hong et al. [139]	PSPT *	Grade 3	5 daily doses of 5 GyE in 1 week	NA	
Gastritis					
Merchant et al. [140]	PSPT	Grade 3	54 GyRBE	NA	NA
Rectal bleeding					
Colaco et al. [141]	PSPT (single field prostate)	Grade 3	78 GyRBE (median)	Prostate (high- and low-risk patients): 78 GyRBE Prostate and proximal seminal vesicles (intermediate-risk patients): dose escalation 78 to 82 GyRBE Dose constraints: Rectal wall: V70 < 30%, V50 < 50% Bladder wall: V82 < 7 cm^3^, V80 < 8 cm^3^,V30 < 35 cm^3^	Rectum: V70 < 20% V50 < 50% Bladder: V80 < 15%
Bryant et al. [142]	PSPT	Grade 3	74–82 Gy at 2 Gy/fr	NA	
Pugh et al. [143]	PSPT or PBS IMPT	Grade 3	75.6–78 Gy	NA	
Elevated biliary enzymes/bilirubin					
Sumiya et al. [145]	PSPT	Grade 2–3	66 Gy/10 fr 72.6 Gy/22 fr 74 Gy/37 fr	NA	
Kawashima et al. [147]	PSPT	Grade 3	72 GyE/16 fr (median)	Percent of liver noncancerous regions receiving ≥30 GyE exceeded 25%	Liver: <30–32 Gy (mean)
Bush et al. [148]	PSPT	Grade 3: 2 cases, Grade 4: 1 case	70.2 Gy/15 fr	No more than 33% liver received 30 Gy	Liver: <30–32 Gy (mean)
Radiation-induced liver disease					
Cheng et al. [146]	PSPT	NA	96.56 GyRBE (median)	NA	
Hepatic insufficiency					
Kawashima et al. [147]	PSPT	Grade 5	72 GyE/16 fr (median)	Percent of liver noncancerous regions receiving ≥30 GyE exceeded 25%	Liver: <30–32 Gy (mean)
Hepatic failure					
Hashimoto et al. [149]	PSPT	Grade 4	1st course: 72 Gy/16 fr (median) following courses: 66 Gy/16 fr (median)	NA	

PBT: proton beam therapy; CRT: chemoradiation therapy; PSPT: passively scattered proton therapy; PT: proton therapy; * treatment may include chemotherapy; ^†^ treatment included systemic therapy; ^‡^ reirradiation.

**Table 5:** Summary of hematological toxicities reported following proton radiation.

Paper	Type of PBT	Toxicity Grade	Prescribed Dose	Dose to Structure/OARs
Leukopenia				
Vennarini et al. [151]	PBS (CSI) *	Grade 3–4	54 Gy (median)	36 Gy (median CSI dose)
Barney et al. [154]	PSPT (CSI) *	Grade 3	54 Gy (median)	30.6 Gy (median CSI dose)
Yang et al. [68]	PBS (hypofractionated CSI)	Grade 3	30 GyRBE in 10 fr	NA
Liu et al. [155]	PSPT (CSI)	Grade 4	54 Gy (median)	23.4 Gy (median CSI dose)
Neutropenia				
Vennarini et al. [151]	PBS (CSI)	Grade 3–4	54 Gy (median)	36 Gy (median CSI dose)
Oshiro et al. [102]	PSPT *	Grade 3–4	74 Gy in 37 fr (primary site) 66 Gy in 33 fr (lymph nodes)	NA
Yegya-Raman et al. [92]	PBS or PSPT	Grade 3: 2, Grade 4: 1	60–70 Gy in fractions	Lung: 6.9 Gy (mean) Heart: 2.5 Gy (mean) Esophagus: 9.7 Gy (mean)
Weinberg et al. [152]	PBS (hypofractionated) *	Grade 3	5 Gy × 5 fractions	NA
Lymphopenia				
Routman et al. [153]	PBS *	Grade 4	50 Gy (median)	NA
Yang et al. [68]	PBS (hypofractionated CSI)	Grade 4	30 GyRBE in 10 fr	NA
Liu et al. [155]	PSPT (Proton CSI)	Grade 4	54 Gy (median)	23.4 Gy (median CSI dose)
Yegya-Raman et al. [92]	PBS or PSPT	Grade 3: 2, Grade 4: 1	60–70 Gy in fractions	Lung: 6.9 Gy (mean) Heart: 2.5 Gy (mean) Esophagus: 9.7 Gy (mean)
Anemia				
Barney et al. [154]	PSPT (CSI) *	Grade 2	54 Gy (median)	30.6 Gy (median CSI dose)
Yegya-Raman et al. [92]	PBS or PSPT	Grade 1: 3	60–70 Gy in fractions	Lung: 6.9 Gy (mean) Heart: 2.5 Gy (mean) Esophagus: 9.7 Gy (mean)
Yang et al. [68]	PBS (hypofractionated CSI)	Grade 3	30 GyRBE in 10 fr	NA
Liu et al. [155]	PSPT (CSI)	Grade 3	54 Gy (median)	23.4 Gy (median CSI dose)
Thrombocytopenia				
Barney et al. [154]	PSPT (CSI) *	Grade 3–4	54 Gy (median)	30.6 Gy (median CSI dose)
Oshiro et al. [102]	PSPT *	Grade 3	74 Gy in 37 fr (primary site) 66 Gy in 33 fr (lymph nodes)	NA
Yang et al. [68]	PBS (hypofractionated CSI)	Grade 4	30 GyRBE in 10 fr	NA
Leukocytopenia				
Oshiro et al. [102]	PBT *	Grade 3	74 Gy in 37 fr (primary site) 66 Gy in 33 fr (lymph nodes)	NA

PBT: proton beam therapy; CSI: craniospinal irradiation; PBS: pencil beam scanning; * treatment may include chemotherapy. Note: no recommendations were made by QUANTEC on bone marrow dose constraints.

**Table 6:** Summary of urological toxicities reported following proton radiation.

Paper	Type of PBT	Toxicity Grade	Prescribed Dose	Dose to Structure/OARs	QUANTEC Photon 3DCRT Dose Constraints
Urinary retention					
Takaoka et al. [156]	PBS (NFPT or MHPT)	Grade 1–2	76–78 Gy in 38–39 fr or 60–63 Gy in 20–21 fr	12.3 Gy or 11.1 Gy (median bladder dose)	Bladder: <65 Gy (max)
Hematuria					
Takaoka et al. [156]	PBS (NFPT or MHPT)	Grade 1–2	76–78 Gy in 38–39 fr or 60–63 Gy in 20–21 fr	12.3 Gy or 11.1 Gy (median bladder dose)	Bladder: <65 Gy (max)
Makishima et al. [157]	PSPT	Grade 3	Low risk: 74 Gy/37 fr Intermediate or high risk: 78 Gy/39 fr	Median rectal V30: 32.5%, V80: 12.5% of prescribed dose	Rectum: V50 < 50% V75 < 15%
Bryant et al. [142]	PSPT	Grade 3–4	74–82 Gy at 2 Gy/fr	NA	
Urinary frequency					
Takaoka et al. [156]	PBS (NFPT or MHPT)	Grade 1–2	76–78 Gy in 38–39 fr or 60–63 Gy in 20–21 fr	12.3 Gy or 11.1 Gy (median bladder dose)	Bladder: <65 Gy (max)
Makishima et al. [157]	PSPT	Grade 2	Low risk: 74 Gy/37 fr Intermediate or high risk: 78 Gy/39 fr	Median rectal V30: 32.5%, V80: 12.5% of prescribed dose	Rectum: V50 < 50% V75 < 15%
Meixner et al. [161]	PBS *	<Grade 3	50.4 Gy (median) in 25–28 fr	NA	
Urinary urgency					
Takaoka et al. [156]	PBS (NFPT or MHPT)	Grade 1–2	76–78 Gy in 38–39 fr or 60–63 Gy in 20–21 fr	12.3 Gy or 11.1 Gy (median bladder dose)	Bladder: <65 Gy (max)
Urinary incontinence					
Takaoka et al. [156]	PBS (NFPT or MHPT)	Grade 1–2	76–78 Gy in 38–39 fr or 60–63 Gy in 20–21 fr	12.3 Gy or 11.1 Gy (median bladder dose)	Bladder: <65 Gy (max)
Hoppe et al. [160]	PSPT		76–82 CGE or 70–72.5 CGE	≥40 CGE (mean penile bulb dose)	Penile bulb: D90 < 50 Gy
Meixner et al. [161]	PBS *	<Grade 3	50.4 Gy (median) in 25–28 fr	NA	
Urinary tract pain					
Takaoka et al. [156]	PBS (NFPT or MHPT)	Grade 1–2	76–78 Gy in 38–39 fr or 60–63 Gy in 20–21 fr	12.3 Gy or 11.1 Gy (median bladder dose)	Bladder: <65 Gy (max)
Non-infectious cystitis					
Makishima et al. [157]	PSPT	Grade 3	Low risk: 74 Gy/37 fr Intermediate or high risk: 78 Gy/39 fr	Median rectal V30: 32.5%, V80: 12.5% of prescribed dose	Rectum: V50 < 50% V75 < 15%
Bladder irritation					
Bryant et al. [142]	PSPT	Grade 3	74–82 Gy at 2 Gy/fr	NA	
Urinary obstruction					
Bryant et al. [142]	PSPT	Grade 3	74–82 Gy at 2 Gy/fr	NA	
Bowel perforation					
Uezono et al. [158]	PSPT (preoperative PBT) *	Grade 3	50.4 Gy	NA	
Erectile Dysfunction					
Chiang et al. [159]	PBS *		50 GyE/25 fr Boost: 78 Gy/39 fr	50.9 Gy (max dose to small bowel)	Small bowel: Individual loops: V15 < 120 cc Peritoneal cavity: V45 < 195 cc
Hoppe et al. [160]	PSPT		76–82 CGE or 70–72.5 CGE	≥40 CGE (mean penile bulb dose)	Penile bulb: D90 < 50 Gy
Nocturia					
Meixner et al. [161]	PBS *	<Grade 3	50.4 Gy (median) in 25–28 fr	NA	
Vaginal bleeding					
Meixner et al. [161]	PBS *	<Grade 3	50.4 Gy (median) in 25–28 fr	NA	
Vaginal mucosal toxicity					
Li et al. [162]	PSPT *^,†^	Grade 1–2	NA	Rectum: 63.8 Gy Bladder: 58 Gy	Rectum: V50 < 50% V75 < 15% Bladder: <65 Gy (max)
Vaginal dryness					
Barcellini et al. [163]	PBS ^†^	Grade 1	39 Gy/13 fr	NA	
Premature ovarian deficiency					
Uezono et al. [158]	PSPT (preoperative PBT) *	Grade 2	50.4 Gy	NA	

NFPT: normo-fractionated proton therapy; MHPT: moderately hypofractionated proton therapy; PBT: proton beam therapy; PSPT: passively scattered proton therapy * treatment may include chemotherapy; ^†^ reirradiation.

**Table 7:** Summary of osteological toxicities reported following proton irradiation.

Paper	Type of PBT	Toxicity Grade	Prescribed Dose	Dose to Structure/OARs	QUANTEC Photon 3DCRT Dose Constraints
Osteoradionecrosis					
Singh et al. [165]	USPT or PBS *	Grade 2–3	66 Gy (median)	ORN site Average Dmean: 71.5 Gy Average Dmax: 75.1 Gy	Mandible: ≤70 Gy (max) Femoral heads: V50 < 5%
Zhang et al. [166]	PBS IMPT *	Grade 1	25.6 Gy (mean)	Mandible: >50 Gy	Mandible: ≤70 Gy (max) Femoral heads: V50 < 5%
Unequal limb length					
Uezono et al. [158]	PSPT (postoperative PBT) *	Grade 2	54 Gy	NA	
Hip dislocation					
Uezono et al. [158]	PSPT (postoperative PBT) *	Grade 3	50.4 Gy	NA	
Rib fracture					
Chang et al. [94]	PSPT (dose-escalated hypofractionated PBT)	Grade 2	87.5 GyRBE in 35 fr (2.5 GyRBE/fr)	Dose limit to bronchial tree and large blood vessels: 87.5 Gy < 10 cm^3^ Dose limit to heart: 70 Gy < 10%	Heart: V25 < 10% Mandible: ≤70 Gy (max) Femoral heads: V50 < 5%
Bush et al. [167]	Hypofractionated PSPT	Grade 2	Dose escalation: 51 to 60 to 70 Gy in 10 fr	NA	

USPT: uniform scanning proton therapy; PBS: pencil beam scanning; IMPT: intensity-modulated proton therapy; PBT: proton beam therapy; PSPT: passively scattered proton therapy; * treatment may include chemotherapy.

**Table 8:** Summary of oral-health-related toxicities reported following proton radiation.

Paper	Type of PBT	Toxicity Grade	Prescribed Dose	Dose to Structure/OARs	QUANTEC Photon 3DCRT Dose Constraints
Microdontia					
Foster-Thomas et al. [168]	PSPT *^,†^	NA	50.4 Gy in 28 fr	Maxilla: 30 Gy (mean) Mandible: 25.9 Gy (mean)	Mandible ≤ 70 Gy (max)
Hypodontia					
Foster-Thomas et al. [168]	PSPT *^,†^	NA	50.4 Gy in 28 fr	Maxilla: 30 Gy (mean) Mandible: 25.9 Gy (mean)	Mandible ≤ 70 Gy (max)
Hypermineralization					
Foster-Thomas et al. [168]	PSPT *^,†^	NA	50.4 Gy in 28 fr	Maxilla: 30 Gy (mean) Mandible: 25.9 Gy (mean)	Mandible ≤ 70 Gy (max)
Abrupt root development					
Foster-Thomas et al. [168]	PSPT *^,†^	NA	50.4 Gy in 28 fr	Maxilla: 30 Gy (mean) Mandible: 25.9 Gy (mean)	Mandible ≤ 70 Gy (max)
Partial/lack of eruption of teeth					
Foster-Thomas et al. [168]	PSPT *^,†^	NA	50.4 Gy in 28 fr	Maxilla: 30 Gy (mean) Mandible: 25.9 Gy (mean)	Mandible ≤ 70 Gy (max)
Xerostomia					
Bagley et al. [170]	PBS IMPT *	NA	69.3 Gy (median)	Mean doses: Parotid: 27.8 ± 5.7 Gy Submandibular: 39.2 ± 21.9 Gy Contralateral parotid: 17.5 ± 9.0 Gy Contralateral submandibular: 39.2 ± 21.9 Gy Oral cavity: 22.4 ± 10.6 Gy	Mandible ≤ 70 Gy (max)
Cao et al. [171]	PBS IMPT *	NA	With chemotherapy: CTV1: 70 Gy CTV2: 63 Gy CTV3: 57 Gy Without chemotherapy: CTV1: 66 Gy CTV2: 60 Gy CTV3: 54 Gy	Dose constraints: Brain: <60 Gy (max) Brainstem and optical apparatus: <54 Gy (max) Spinal cord: <45 Gy (max) Cornea, cochlea: <35 Gy (max) Larynx: <35 Gy (mean) Oral cavity: <30 Gy (mean) Salivary glands: <26 Gy (mean)	Parotid (bilateral) ≤ 25 Gy (mean) Mandible ≤ 70 Gy (max)
Chuong et al. [172]	PBS or USPT *	Grade 2	66.5 GyE (median) in 33 fr	Dose constraints: Optic nerve/chiasm: 54 Gy (max) Spinal cord and brainstem: <45 Gy (max) Larynx: <45 Gy (mean) Oral cavity: <30 Gy (mean) Contralateral submandibular: <20 Gy (mean) Contralateral parotid: <10 Gy (mean)	Parotid bilateral ≤ 25 Gy (mean) Mandible ≤ 70 Gy (max)
Dysgeusia					
Chuong et al. [172]	PBS or USPT *	Grade 2	66.5 GyE (median) in 33 fr	Dose constraints: Optic nerve/chiasm: 54 Gy (max) Spinal cord and brainstem: <45 Gy (max) Larynx: <45 Gy (mean) Oral cavity: <30 Gy (mean) Contralateral submandibular: <20 Gy (mean) Contralateral parotid: <10 Gy (mean)	Parotid bilateral ≤ 25 Gy (mean) Mandible ≤ 70 Gy (max)
Dysphagia					
Chuong et al. [172]	PBS or USPT *	Grade 2	66.5 GyE (median) in 33 fr	Dose constraints: Optic nerve/chiasm: 54 Gy (max) Spinal cord and brainstem: <45 Gy (max) Larynx: <45 Gy (mean) Oral cavity: <30 Gy (mean) Contralateral submandibular: <20 Gy (mean) Contralateral parotid: <10 Gy (mean)	Parotid bilateral ≤ 25 Gy (mean) Mandible ≤ 70 Gy (max) Esophagus < 34 Gy (mean)
Chuong et al. [172]	PBS or USPT *	Grade 3	66.5 GyE (median) in 33 fr	Dose constraints: Optic nerve/chiasm: 54 Gy (max) Spinal cord and brainstem: <45 Gy (max) Larynx: <45 Gy (mean) Oral cavity: <30 Gy (mean) Contralateral submandibular: <20 Gy (mean) Contralateral parotid: <10 Gy (mean)	Parotid bilateral ≤ 25 Gy (mean) Mandible ≤ 70 Gy (max) Esophagus < 34 Gy (mean)
Oral mucositis					
Chuong et al. [172]	PBS or USPT *	Grade 2	66.5 GyE (median) in 33 fr	Dose constraints: Optic nerve/chiasm: 54 Gy (max) Spinal cord and brainstem: <45 Gy (max) Larynx: <45 Gy (mean) Oral cavity: <30 Gy (mean) Contralateral submandibular: <20 Gy (mean) Contralateral parotid: <10 Gy (mean)	Parotid bilateral ≤ 25 Gy (mean) Mandible ≤ 70 Gy (max)
Chuong et al. [172]	PBS or USPT *	Grade 3	66.5 GyE (median) in 33 fr	Dose constraints: Optic nerve/chiasm: 54 Gy (max) Spinal cord and brainstem: <45 Gy (max) Larynx: <45 Gy (mean) Oral cavity: <30 Gy (mean) Contralateral submandibular: <20 Gy (mean) Contralateral parotid: <10 Gy (mean)	Parotid bilateral ≤ 25 Gy (mean) Mandible ≤ 70 Gy (max)

PBS: pencil beam scanning; USPT: uniform scanning proton therapy; PBT: proton beam therapy; IMPT: intensity-modulated proton therapy; CTV: clinical target volume; * treatment may include chemotherapy; ^†^ same patient.

**Table 9:** Summary of endocrine toxicities reported following proton radiation.

Paper	Type of PBT	Toxicity Grade	Prescribed Dose	Dose to Structure/OARs
Endocrine deficits				
Sanford et al. [61]	PSPT or PBS	Grade 2: 6 patients (55.8 Gy dose) Grade 4: 1 patient (55.8 Gy dose) Grade 2: 10 patients (63.0 Gy dose)	55.8 GyRBE or 63.0 GyRBE	NA
Indelicato et al. [54]	PSPT	Grade 2+: 13 patients	59.4 GyRBE (patients > 3 years old) 54 GyRBE (patients ≤ 3 years old)	NA
Growth hormone deficiency				
Greenfield et al. [33]	PSPT or PBS *	1 patient unspecified grade with hypothyroidism, 7 patients with unspecified grade	52.2 GyRBE (median)	38 Gy (hypothalamic–pituitary mean dose)
Ladra et al. [75]	PSPT *	Grade 2: 3 patients	50.4 GyRBE (median)	NA
Eaton et al. [176]	PSPT	21 patients unspecified grade	54–55.8 Gy as 1.8 Gy/fr	23.4 Gy (median CSI dose)
Panhypopituitarism				
Bishop et al. [175]	PSPT	NA	50.4 GyRBE (median)	NA
Adrenal insufficiency				
Eaton et al. [176]	PSPT	2 patients unspecified grade	54–55.8 Gy as 1.8 Gy/fr	23.4 Gy (median CSI dose)
Shih et al. [71]	PSPT	4 patients unspecified grade	54 GyRBE/30 fr	NA
Sex hormone deficiency				
Eaton et al. [176]	PSPT	1 patient unspecified grade	54–55.8 Gy as 1.8 Gy/fr	23.4 Gy (median CSI dose)
Hypothyroidism				
Eaton et al. [176]	PSPT	9 patients unspecified grade	54–55.8 Gy as 1.8 Gy/fr	23.4 (median CSI dose)
Shih et al. [71]	PSPT	3 patients unspecified grade	54 GyRBE/30 fr	NA
Greenfield et al. [33]	PSPT or PBS *	1 patient unspecified grade with growth hormone deficiency	52.2 GyRBE (median)	38 Gy (hypothalamic–pituitary mean dose)
Hypogonadism				
Shih et al. [71]	PSPT	2 patients unspecified grade	54 GyRBE/30 fr	NA
Greenfield et al. [33]	PSPT or PBS *	1 patient unspecified grade	52.2 GyRBE (median)	48 Gy (hypothalamic–pituitary mean dose)

CSI: craniospinal irradiation; PSPT: passively scattered proton therapy; PBS: pencil beam scanning; * treatment may include chemotherapy.
